# Effect of Tai Chi vs Aerobic Exercise on Blood Pressure in Patients With Prehypertension: A Randomized Clinical Trial

**DOI:** 10.1001/jamanetworkopen.2023.54937

**Published:** 2024-02-09

**Authors:** Xinye Li, Peifen Chang, Min Wu, Yuchen Jiang, Yonghong Gao, Hengwen Chen, Liyuan Tao, Dawei Wei, Xiaochen Yang, Xingjiang Xiong, Yan Yang, Xiandu Pan, Ran Zhao, Fan Yang, Jiahao Sun, Shengjie Yang, Li Tian, Xiaofang He, Eryu Wang, Yiyuan Yang, Yanwei Xing

**Affiliations:** 1 Guang’anmen Hospital, China Academy of Chinese Medical Sciences, Beijing; 2 Graduate School of Beijing University of Chinese Medicine, Beijing, China; 3 Department of Cardiovascular Medicine, Dongzhimen Hospital, Beijing University of Chinese Medicine, Beijing, China; 4 Key Laboratory of Chinese Internal Medicine of the Ministry of Education, Dongzhimen Hospital, Beijing University of Chinese Medicine, Beijing; 5 Clinical Epidemiology Research Center of the Third Hospital of Peking University, Beijing, China; 6 Vasculocardiology Department, Fuzhou Hospital of Traditional Chinese Medicine, Fujian University of Traditional Chinese Medicine, Fuzhou, China; 7 YongDingLu Community Health Care Center, Aerospace Center Hospital, Beijing, China; 8 Traditional Chinese Medicine Department, BaiLi Traditional Chinese Medicine Clinic, Beijing; 9 Medical Department of Beijing Gulou Traditional Chinese Medicine Hospital, Beijing, China; 10 Cardiovascular Department of Affiliated Hospital of Shanxi University of Chinese Medicine, Taiyuan, China

## Abstract

**Importance:**

Prehypertension increases the risk of developing hypertension and other cardiovascular diseases. Early and effective intervention for patients with prehypertension is highly important.

**Objective:**

To assess the efficacy of Tai Chi vs aerobic exercise in patients with prehypertension.

**Design, Setting, and Participants:**

This prospective, single-blinded randomized clinical trial was conducted between July 25, 2019, and January 24, 2022, at 2 tertiary public hospitals in China. Participants included 342 adults aged 18 to 65 years with prehypertension, defined as systolic blood pressure (SBP) of 120 to 139 mm Hg and/or diastolic BP (DBP) of 80 to 89 mm Hg.

**Interventions:**

Participants were randomized in a 1:1 ratio to a Tai Chi group (n = 173) or an aerobic exercise group (n = 169). Both groups performed four 60-minute supervised sessions per week for 12 months.

**Main Outcomes and Measures:**

The primary outcome was SBP at 12 months obtained in the office setting. Secondary outcomes included SBP at 6 months and DBP at 6 and 12 months obtained in the office setting and 24-hour ambulatory BP at 12 months.

**Results:**

Of the 1189 patients screened, 342 (mean [SD] age, 49.3 [11.9] years; 166 men [48.5%] and 176 women [51.5%]) were randomized to 1 of 2 intervention groups: 173 to Tai Chi and 169 to aerobic exercise. At 12 months, the change in office SBP was significantly different between groups by −2.40 (95% CI, −4.39 to −0.41) mm Hg (*P* = .02), with a mean (SD) change of −7.01 (10.12) mm Hg in the Tai Chi group vs −4.61 (8.47) mm Hg in the aerobic exercise group. The analysis of office SBP at 6 months yielded similar results (−2.31 [95% CI, −3.94 to −0.67] mm Hg; *P* = .006). Additionally, 24-hour ambulatory SBP (−2.16 [95% CI, −3.84 to −0.47] mm Hg; *P* = .01) and nighttime ambulatory SBP (−4.08 [95% CI, −6.59 to −1.57] mm Hg; *P* = .002) were significantly reduced in the Tai Chi group compared with the aerobic exercise group.

**Conclusions and Relevance:**

In this study including patients with prehypertension, a 12-month Tai Chi intervention was more effective than aerobic exercise in reducing SBP. These findings suggest that Tai Chi may help promote the prevention of cardiovascular disease in populations with prehypertension.

**Trial Registration:**

Chinese Clinical Trial Registry Identifier: ChiCTR1900024368

## Key Points

**Question:**

Is Tai Chi more effective in reducing blood pressure (BP) for patients with prehypertension compared with aerobic exercise?

**Findings:**

In this randomized clinical trial that included 342 participants, the mean decrease in systolic BP from baseline to month 12 was significantly greater in the Tai Chi group compared with the aerobic exercise group.

**Meaning:**

Among patients with prehypertension, Tai Chi was shown to be more effective than aerobic exercise in reducing BP after 12 months.

## Teaser

This randomized clinical trial assesses whether Tai Chi is more effective than aerobic exercise in reducing blood pressure among patients with prehypertension.

## Introduction

Prehypertension, defined as a systolic blood pressure (SBP) of 120 to 139 mm Hg and/or a diastolic BP (DBP) of 80 to 89 mm Hg, was introduced by the seventh report of the Joint National Committee on Prevention, Detection, Evaluation, and Treatment of High Blood Pressure in 2003.^1^ According to the results of the Chinese Adult Hypertension Survey published in 2021,^2^ approximately 50.9% of adults without a history of hypertension had prehypertension. Prehypertension is associated with an increased risk of hypertension, cardiovascular disease, coronary heart disease, cerebrovascular disease, and myocardial infarction.^3,4,5^ Moreover, prehypertension is associated with target organ damage, such as early arteriosclerosis and left ventricular dysfunction.^6,7,8^ Therefore, patients with prehypertension should receive early and effective intervention and treatment to adjust the prevention strategy and reduce the occurrence of future hypertension and organ damage.

Increasing evidence suggests that exercise interventions reduce BP in individuals with hypertension or prehypertension.^9,10,11^ The guidelines recommend exercise as a priority in treating and managing populations with prehypertension, as exercise can improve their lifestyles and is potentially cost-effective compared with antihypertensives.^12,13^ Although aerobic exercise is recommended to reduce BP,^14^ it has some limitations, such as the need for space and possible risk of joint damage. Traditional Chinese exercise, Tai Chi, is a mind-body exercise that benefits balance and cardiovascular and respiratory function.^10,15,16^ Studies have shown that Tai Chi can effectively reduce BP after 12 weeks, 9 months, or 12 months of intervention.^10,17,18,19^ As a low-impact, enjoyable, and economic form of exercise, Tai Chi is expected to be a viable substitute exercise mode for aerobic exercise for reducing BP and compensates for the limitations of aerobic exercise to a certain extent. However, evidence is scarce as to whether Tai Chi is superior to aerobic exercise in reducing BP in patients with prehypertension. This randomized clinical trial used a rigorous design to test effectiveness of Tai Chi and aerobic exercise in reducing BP in this population.

## Methods

### Study Design and Settings

We performed a single-blind, parallel-design, randomized clinical trial in 2 tertiary public hospitals (Guang’anmen Hospital of China Academy of Chinese Medical Sciences and Dongzhimen Hospital of Beijing University of Chinese Medicine) in Beijing, China. Participants were randomly assigned to the Tai Chi training program (Tai Chi group) or the moderate-intensity aerobic exercise training program (aerobic exercise group). The study design has been published previously.^20^ The institutional review board at each site approved the study protocol (found in Supplement 1). This study followed the Consolidated Standards of Reporting Trials (CONSORT) reporting guideline. Written informed consent was obtained from all participants.

### Participants and Recruitment

Participants were recruited through flyers posted at local community centers, medical clinics, and targeted online media advertisements. Participants were considered eligible for this study if they (1) were aged 18 to 65 years; (2) fulfilled the classification of prehypertension with an SBP in the range of 120 to 139 mm Hg and/or a DBP in the range of 80 mm Hg to 89 mm Hg^1^; (3) had not been treated with western medicine or traditional Chinese medicine, acupuncture, and moxibustion for BP management (or the treatment was discontinued for 2 weeks); (4) were willing to be randomized to the Tai Chi group or the aerobic exercise group; (5) had the ability to complete written questionnaires and operate electronic equipment independently; and (6) were able to give informed consent. We excluded individuals with type 1 or 2 diabetes, coronary heart disease, chronic kidney disease (estimated glomerular filtration rate <60 mL/min), or Shy-Drager syndrome or who were pregnant or lactating (detailed criteria for eligibility are provided in eMethods in Supplement 2).

### Randomization and Blinding

Participants were randomized (1:1) to receive either Tai Chi or aerobic exercise via a central web-based randomization system. To ensure the concealment of allocation, a 24-hour central web-based automated randomization system was adopted for all randomization processes, using the static random method and the SAS, version 9.4 software PROC PLAN process programming (SAS Institute Inc). When randomization was complete, the outcome assessors who evaluated the effects of the treatments received only the participant number and interpreted the data blinded to group allocation.

### Interventions

Patients in both groups underwent a 12-month Tai Chi or aerobic exercise training program 4 times weekly. In both groups, each session consisted of a 10-minute warm-up, 40 minutes of core exercises, and a 10-minute cool-down activity. The 24-form Yang-style Tai Chi, consisting of 24 standard movements, was adopted for the Tai Chi intervention. Aerobic exercise interventions included climbing stairs, jogging, brisk walking, and cycling. Exercise intensity in the aerobic exercise group was monitored. The maximum heart rate was estimated as 208 − (0.7 × age in years).^21^ At each site, the 2 interventions were separated in time to avoid cross-contamination. Participants performed the exercises collectively no less than 1 time per week and used uploaded videos 3 times per week. Participants were required to sign in to confirm accurate attendance records, whether they attended the session or practiced at home. Throughout the study, all sessions were regularly monitored and received feedback to ensure proper instruction. To maximize the replicability of interventions, the exercise program was comprehensively described following the Template for Intervention Description and Replication checklist (eTable 1 in Supplement 2). The intervention fidelity was determined by required qualifications of the instructors and instruction completed during the sessions, the implementation of intervention, and participant adherence (eTable 2 in Supplement 2).

### Outcomes

The study outcome measures were assessed at baseline and 6 and 12 months (at the end of the intervention). The primary outcome measure was the mean change in SBP in the office setting from baseline to 12 months. Secondary outcomes included mean changes in SBP in the office setting at 6 months; mean changes in DBP in the office setting at 6 and 12 months; mean changes in 24-hour ambulatory BP (24-hour ambulatory SBP, 24-hour ambulatory DBP, daytime ambulatory SBP, daytime ambulatory DBP, nighttime ambulatory SBP, and nighttime ambulatory DBP), lipid profile (low-density lipoprotein cholesterol, high-density lipoprotein cholesterol, total cholesterol, and triglyceride levels), metabolic parameters (fasting plasma glucose, glycated hemoglobin, and creatinine levels), Medical Outcomes Study 36-item Short-Form Health Survey, Systematic Coronary Risk Estimation (SCORE), waist circumference, weight, body mass index at 12 months, and adverse events (eg, being hospitalized or death of any cause), including adverse effects during or after the exercise sessions (eg, severe hypotension). Other assessments included the mean daily caloric intake over the last 7 days, the 1-week total physical activity, adherence (assessed via Tai Chi or aerobic exercise sessions), and safety evaluations. A detailed description of the assessment procedures is provided in eMethods in Supplement 2.

### Statistical Analysis

The primary outcome of this study was the change in office SBP at 12 months. Sample size was chosen based on the comparison of the office SBP reduction among individuals in the Tai Chi group and the aerobic exercise group. Based on the mean reduction of SBP in the studies conducted before the start of the trial,^10,22^ we hypothesized that the SBP in the Tai Chi group would be reduced by 4.6 mm Hg more than in the aerobic exercise group. We further conservatively assumed an SD of 13.4 in both groups. To have 80% power at a 2-sided α level of .05 and under the assumption of a loss of follow-up of 20%, the study required 342 patients in total.

According to our study protocol, an interim analysis regarding trial progression was performed after 12-month follow-up of the first 171 participants, corresponding to 50% of the study population. We used the O’Brien-Fleming adjusted level α = .005.^23^ There were no differences in office SBP, as primary outcome measure, between the groups (*P* = .07 [greater than the O’Brien-Fleming adjusted level α). The independent data monitoring committee recommended continuing the trial as planned. The nominal α level for the primary outcome in the final analysis was equal to .049 due to the interim analysis.

All efficacy analyses were performed in the intention-to-treat population. We reported continuous variables as mean (SD) or median (IQR). We used 1-way analysis of variance or the Kruskal-Wallis test for between-group comparisons as appropriate. Categorical variables were described using numbers and percentages and analyzed using the χ^2^ test or the Fisher exact test.

Unpaired *t* tests were performed to examine between-group differences at baseline and in mean change from baseline to 6 and 12 months in primary and secondary outcomes. Paired *t* tests were performed for within-group comparisons from baseline to 6 and 12 months, and results were reported as mean difference and 95% CI. Analysis of covariance was also used to adjust for baseline BP measurements. The follow-up time, mean daily calories, and total physical activity were compared using the Wilcoxon-Mann-Whitney test, and the median difference was presented as an effect measure with 95% CI estimated using the bootstrap method with 1000 replicates. The multiple imputation method was preferred for analyzing the missing data. The participants’ demographic characteristics (age and sex) were used as the predictor variables for imputed data. Given the large number of secondary outcomes, the secondary outcomes should be interpreted as exploratory. All statistical analyses were performed using SPSS, version 24.0 (IBM Corporation), with a 2-sided *P* < .05 considered statistically significant.

## Results

### Participants

**Figure 1.**
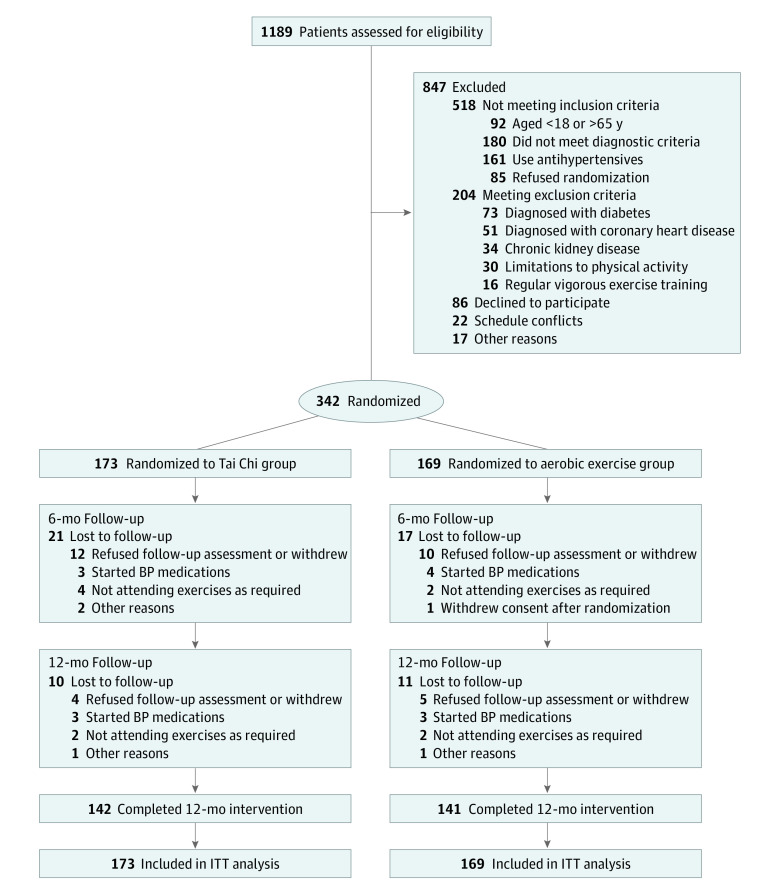
Study Flow Diagram Diabetes includes type 1 or type 2. BP indicates blood pressure; ITT, intention-to-treat.

A total of 342 participants (mean [SD] age, 49.3 [11.9] years; 166 [48.5%] men and 176 [51.5%] women; all Chinese) were randomized to the Tai Chi group (n = 173) and aerobic exercise group (n = 169) and included in the intention-to-treat analysis. This randomized clinical trial was conducted between July 25, 2019, and January 24, 2022, at 2 tertiary public hospitals in China. Among the 1189 patients screened, 342 met the enrollment criteria and consented to participate. Fifty-nine patients (17.3%) discontinued participation in the study; thus, 283 (82.7%) completed the follow-up assessments and were included in the analysis (Figure 1). The baseline demographic and clinical characteristics of participants are summarized in Table 1. eTable 3 in Supplement 2 shows the baseline characteristics of participants between the 2 centers. eTable 4 in Supplement 2 shows the differences in demographic characteristics, BP, and SCORE risk between patients who completed the study and those who dropped out. The median follow-up time of the Tai Chi group was 12.00 (IQR, 11.93-12.13) months; that of the aerobic exercise group, 12.00 (IQR, 11.77-12.15) months (eTable 5 in Supplement 2). There were no significant differences in the follow-up time between the groups (*P* = .13).

**Table 1.** Participant Characteristics at Baseline

Characteristic	Tai Chi group (n = 173)	Aerobic exercise group (n = 169)
Age, mean (SD), y	48.4 (12.4)	50.1 (11.4)
Sex, No. (%)		
Women	87 (50.3)	89 (52.7)
Men	86 (49.7)	80 (47.3)
Weight, mean (SD), kg	71.2 (12.4)	69.2 (11.7)
Body mass index, mean (SD)^a^	25.5 (3.3)	24.8 (3.2)
Waist circumference, mean (SD), cm	88.8 (9.3)	87.1 (9.4)
Medical history, No. (%)		
Current smoker	18 (10.4)	21 (12.4)
Previous smoker	4 (2.3)	9 (5.3)
Hyperlipidemia	29 (16.8)	39 (23.1)
Family history of hypertension	121 (69.9)	117 (69.2)
Family history of coronary artery disease	62 (35.8)	49 (29.0)
SCORE risk, mean (SD)^b^	0.82 (1.23)	1.07 (1.62)
Office BP, mean (SD), mm Hg		
SBP	132.4 (6.0)	132.6 (6.0)
DBP	84.2 (4.9)	84.5 (4.7)
Ambulatory BP, mean (SD), mm Hg		
24-h SBP	128.8 (9.5)	128.2 (9.4)
24-h DBP	81.7 (8.0)	81.5 (8.6)
Daytime SBP	131.5 (9.4)	130.9 (9.5)
Daytime DBP	83.2 (8.3)	82.8 (9.0)
Nighttime SBP	123.9 (12.0)	122.6 (14.7)
Nighttime DBP	78.9 (10.2)	78.7 (11.1)
Fasting plasma glucose level, mg/dL	101.8 (16.4)	102.0 (24.1)
HbA_1c_ level, %	5.70 (0.46)	5.70 (0.50)
Total cholesterol level, mg/dL	197.3 (38.6)	199.6 (37.5)
Triglyceride level, mg/dL	155.8 (115.9)	155.8 (207.1)
LDL cholesterol level, mg/dL	121.6 (29.3)	120.8 (26.3)
HDL cholesterol level, mg/dL	50.6 (9.7)	53.9 (13.5)
Creatinine level, mg/dL	0.72 (0.14)	0.72 (0.14)

Abbreviations: DBP, diastolic blood pressure (BP); HbA_1c_, glycated hemoglobin; HDL, high-density lipoprotein; LDL, low-density lipoprotein; SBP, systolic BP; SCORE, Systematic Coronary Risk Evaluation.

SI conversion factors: To convert cholesterol to mmol/L, multiply by 0.0259; creatinine to μmol/L, multiply by 88.4; glucose to mmol/L, multiply by 0.0555; and triglyceride to mmol/L, multiply by 0.0113.

^a^ Calculated as weight in kilograms divided by height in meters squared.

^b^ The SCORE system estimates an individual’s 10-year risk of fatal cardiovascular disease. Risks range from 0 to 14 in women and 0 to 26 in men.

### Primary Outcomes

After 12 months, the mean change in office SBP was significantly different between groups by −2.40 (95% CI, −4.39 to −0.41) mm Hg (*P* = .02). The mean (SD) change in office SBP was −7.01 (10.12) mm Hg in the Tai Chi group vs −4.61 (8.47) mm Hg in the aerobic exercise group (Table 2).

**Table 2.** Changes in Office Blood Pressure at Baseline and After 6- and 12-Month Interventions

Outcome	Study group, mean (SD)		Mean between-group difference in change (95% CI)	*P* value
	Tai Chi group (n = 173)	Aerobic exercise group (n = 169)		
Office SBP at baseline, mm Hg	132.4 (6.0)	132.6 (6.0)	NA	.74
Mean change in office SBP, mm Hg				
6 mo	−6.18 (8.00)^a^	−3.88 (7.30)^a^	−2.31 (−3.94 to −0.67)	.006
12 mo	−7.01 (10.12)^a^	−4.61 (8.47)^a^	−2.40 (−4.39 to −0.41)	.02
Office DBP at baseline, mm Hg	84.2 (4.9)	84.5 (4.7)	NA	.58
Mean change in office DBP, mm Hg				
6 mo	−3.52 (4.23)^a^	−2.60 (4.53)^a^	−0.92 (−1.86 to 0.01)	.052
12 mo	−3.73 (6.21)^a^	−2.56 (6.54)^a^	−1.17 (−2.53 to 0.19)	.09

Abbreviations: DBP, diastolic blood pressure; NA, not applicable; SBP, systolic blood pressure.

^a^ Significantly different from baseline to post intervention (*P* < .001).

### Secondary Outcomes

**Figure 2.**
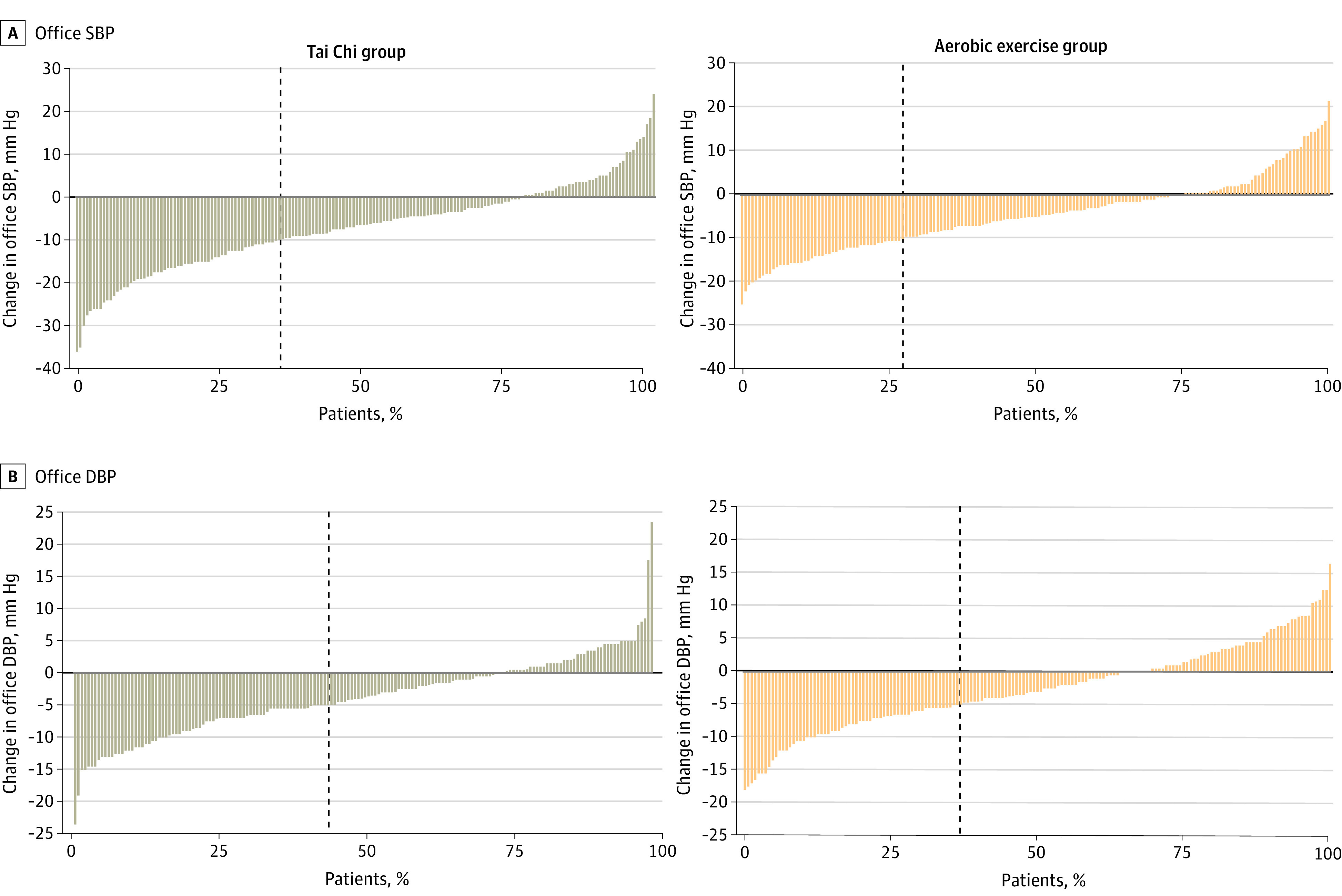
Individual Changes in Office Blood Pressure After 12-Month Intervention With Tai Chi or Aerobic Exercise Vertical dashed lines indicate the percentage of patients with a reduction in office systolic blood pressure (SBP) of at least 10 mm Hg and office diastolic blood pressure (DBP) of at least 5 mm Hg; these percentages were not significantly different between groups (*P* = .14 and *P* = .17, respectively).

After 6 months, the mean reduction in office SBP of the Tai Chi group was greater than that of the aerobic exercise group (−2.31 [95% CI, −3.94 to −0.67] mm Hg; *P* = .006) (Table 2). There were no differences in office DBP between the groups after 6 months (−0.92 [95% CI, −1.86 to 0.01] mm Hg; *P* = .052) (Table 2). After the 12-month intervention, we did not observe significant differences in the mean reduction of office DBP (−1.17 [95% CI, −2.53 to 0.19] mm Hg; *P* = .09), but the Tai Chi group presented with a lower mean (SD) office DBP compared with that of the aerobic exercise group (−3.73 [6.21] vs −2.56 [6.54] mm Hg) (Table 2). After 12 months, 31 of 142 patients (21.8%) from the Tai Chi group showed a BP within reference range (defined as SBP <120 mm Hg and DBP <80 mm Hg, without medications); 22 of 141 patients (15.6%) from the aerobic exercise group showed a BP within reference range (eFigure in Supplement 2). Fewer patients presented with hypertension (defined as SBP >140 mm Hg or DBP >90 mm Hg) in the Tai Chi group than in the aerobic exercise group (17 [12.0%] vs 25 [17.7%]) (eFigure in Supplement 2). Figure 2 shows the individual changes from baseline in office SBP and DBP after 12 months of intervention. Sixty-one patients (35.3%) had an office SBP reduction of at least 10 mm Hg in the Tai Chi group compared with 47 (27.8%) in the aerobic exercise group (*P* = .14) (Figure 2A). Seventy-six patients (43.9%) had an office DBP reduction of at least 5 mm Hg in the Tai Chi group and 62 (36.7%) in the aerobic exercise group (*P* = .17) (Figure 2B).

After the 12-month intervention, the mean reduction in the 24-hour ambulatory SBP of the Tai Chi group was greater than that of the aerobic exercise group (−2.16 [95% CI, −3.84 to −0.47] mm Hg; *P* = .01) (Table 3). Nighttime ambulatory SBP (−4.08 [95% CI, −6.59 to −1.57] mm Hg; *P* = .002) was also significantly reduced in the Tai Chi group compared with the aerobic exercise group (Table 3). There were no differences in 24-hour ambulatory DBP, daytime ambulatory BP, and nighttime ambulatory DBP between the groups. In addition, we observed significant differences in the mean nighttime ambulatory pulse rate of the Tai Chi group compared with that of the aerobic exercise group after 12 months (−2.25 [95% CI, −3.95 to −0.55] beats/min; *P* = .01) (eTable 6 in Supplement 2). There were no differences in 24-hour ambulatory pulse rate and daytime ambulatory pulse rate between the groups. The mean reduction of 24-hour ambulatory SBP load in the Tai Chi group was significantly greater than that in the aerobic exercise group (−6.13% [95% CI, −10.80% to −1.45%]; *P* = .01). Mean nighttime SBP load (−9.67% [95% CI, −15.65% to −3.70%]; *P* = .002) was also significantly reduced in the Tai Chi group compared with the aerobic exercise group. There were no differences in 24-hour DBP load, daytime BP load, and nighttime DBP load between the groups.

**Table 3.** Changes in Ambulatory Blood Pressure at Baseline and After the 12-Month Intervention

Ambulatory measurement	Study group, mean (SD), mm Hg						Mean between-group difference in change (95% CI)	*P* value
	Tai Chi (n = 173)			Aerobic exercise (n = 169)				
	Baseline	12 mo	Change from baseline	Baseline	12 mo	Change from baseline		
24-h SBP	128.8 (9.5)	125.6 (8.2)	−3.20 (8.31)^a^	128.2 (9.4)	127.2 (8.2)	−1.04 (7.48)	−2.16 (−3.84 to −0.47)	.01
24-h DBP	81.7 (8.0)	79.9 (7.0)	−1.75 (7.38)^b^	81.5 (8.6)	80.2 (7.3)	−1.35 (7.07)^c^	−0.40 (−1.94 to 1.13)	.61
Daytime SBP	131.5 (9.4)	128.2 (8.3)	−3.34 (8.21)^a^	130.9 (9.5)	129.3 (8.7)	−1.59 (8.57)^c^	−1.76 (−3.55 to 0.03)	.054
Daytime DBP	83.2 (8.3)	81.2 (7.3)	−1.99 (7.52)^b^	82.8 (9.0)	81.3 (7.7)	−1.55 (7.96)^c^	−0.44 (−2.09 to 1.21)	.60
Nighttime SBP	123.9 (12.0)	120.4 (10.5)	−3.45 (12.04)^a^	122.6 (14.7)	123.2 (10.0)	0.63 (11.50)	−4.08 (−6.59 to −1.57)	.002
Nighttime DBP	78.9 (10.2)	77.5 (7.7)	−1.41 (10.83)	78.7 (11.1)	78.0 (7.6)	−0.70 (9.62)	−0.71 (−2.89 to 1.48)	.53

Abbreviations: DBP, diastolic blood pressure; SBP, systolic blood pressure.

^a^ Significantly different from baseline to post intervention (*P* < .001).

^b^ Significantly different from baseline to post intervention (*P* < .01).

^c^ Significantly different from baseline to post intervention (*P* < .05).

There were no differences in SCORE risk between the groups. We did not observe significant differences in any of the eight 36-item Short-Form Health Survey domains between the 2 groups (eTable 6 in Supplement 2).

### Other Outcomes

There were no between-group differences in waist circumference, weight, body mass index, and biochemical parameters (eTable 7 in Supplement 2). At baseline and 12 months, there was no significant difference in the mean daily caloric intake and total physical activity between the 2 groups (eTable 8 in Supplement 2). The mean (SD) heart rate of the aerobic exercise group was 114.2 (8.25) beats/min. Brisk walking accounted for the highest proportion (48 [34.0%]) of all exercise forms in the aerobic exercise group (eTable 9 in Supplement 2).

### Safety and Compliance

The overall mean attendance rates of the Tai Chi and aerobic exercise groups during the 12 months of intervention were 87.3% and 85.7%, respectively. No serious adverse events or complications were reported during the study.

## Discussion

This randomized clinical trial found that 12 months of Tai Chi significantly decreased office SBP in patients with prehypertension by 2.40 mm Hg more than aerobic exercise. Furthermore, the Tai Chi group showed a greater reduction in 24-hour (−2.16 mm Hg) and nighttime (−4.08 mm Hg) ambulatory SBP than the aerobic exercise group.

As a safe, moderate-intensity, multimodal mind-body exercise, Tai Chi uses a progressive approach that guides individuals to concentrate on slow and fluid movements.^24^ Tai Chi is suitable for people of all ages and physical conditions to practice. From the perspective of implementation, a Tai Chi program is easy to introduce and practice in community settings, thereby providing primary care for populations with prehypertension. Tai Chi can help improve body flexibility, balance, and cardiopulmonary function while reducing the risk of falls.^15,25^ Participants in Tai Chi may receive more group support and hands-on feedback during the process of acquiring a skill. Practicing Tai Chi requires the cooperation of extensive experienced instructors and a period of gradual learning.

Although the trial overlapped with the COVID-19 pandemic, we managed to maintain normal exercise activities, and all patients were given 12 months of exercise intervention. The results of this study are congruent with the findings of recent systematic reviews and meta-analyses on exercise reducing BP.^26,27,28^ Pan et al^29^ conducted a meta-analysis of 24 studies involving 2095 participants to evaluate the BP-lowering effect of Tai Chi among patients with hypertension. The results showed that Tai Chi effectively improved SBP, DBP, and quality of life in patients with hypertension.^29^ Our results on the BP-lowering effect of Tai Chi are comparable to those of previous studies.^10,30^ A study conducted in China has shown that brachial-ankle pulse wave velocity increased by 8.467 cm/s with each 1-mm Hg increment in SBP in elderly patients with hypertension, indicating a higher risk of arterial stiffness.^31^ In our study of patients with prehypertension, Tai Chi was shown to be more effective by −2.40 mm Hg than aerobic exercise in reducing SBP, which may have clinical significance in preventing cardiovascular diseases and even reducing the risk of cardiovascular events.

Our study provides additional important findings. First, the 24-hour ambulatory and nighttime ambulatory SBP of the Tai Chi group were significantly reduced. Second, a significant decrease in nighttime ambulatory pulse rate was observed in the Tai Chi group in our study. One potential explanation is that Tai Chi may play an important role in reducing sympathetic excitability. A previous study^16^ showed that Tai Chi exercise might produce a relaxing effect, enhance vagal modulation, and decrease sympathetic modulation. Third, the SBP load of the Tai Chi group was significantly reduced. Even in participants with normal BP levels, increased BP load can lead to an increased risk of developing hypertension.^32,33^ Twelve months of Tai Chi are superior to aerobic exercise for reducing BP load in patients with prehypertension, which would be more beneficial in reducing the risk of hypertension. Furthermore, when our current randomized study was designed, no refined cardiovascular risk predictions based on different countries or regions existed. Hence, we adhered to the predetermined protocol and maintained consistency using SCORE. We recommend that our results using SCORE to assess cardiovascular risk in China be treated with caution and reservation.

### Strengths and Limitations

The major strengths of our study were that we recruited a relatively large sample of 342 patients from 2 centers, and the study intervention lasted for 12 months, which enhanced scientific merit and persuasiveness. However, the study did not have the ability to detect potential effects in subgroups of interest (eg, comparing different BP stratifications). Secondary outcomes, especially changes in nighttime BP and heart rate, should be understood as exploratory and not be overinterpreted.

## Conclusions

In this randomized clinical trial conducted in office and 24-hour ambulatory conditions, 12 months of Tai Chi was more effective than aerobic exercise in reducing SBP in patients with prehypertension. These findings support the important public health value of Tai Chi to promote the prevention of cardiovascular disease in populations with prehypertension.

## Notes

Supplement 1.
Trial Protocol

Supplement 2. **eMethods.** Criteria for Eligibility, BP Measurement, and Assessments **eTable 1. **Overview of Exercise Intervention, as per TIDieR Criteria **eTable 2.** Intervention Fidelity Checklist **eTable 3.** Baseline Characteristics of Participants Between the 2 Centers **eTable 4. **Assessment of Differences in the Demographics, BP, and SCORE Between the Patients Who Completed the Study and Those Who Dropped Out **eTable 5. **Follow-Up Time of the Tai Chi Group and Aerobic Exercise Group **eTable 6. **Changes in Ambulatory Pulse Rate, Ambulatory Blood Pressure Load, SCORE, and SF-36 After the 12-Month Intervention **eTable 7. **Changes in Body Composition and Biochemical Parameters After the 12-Month Intervention **eTable 8. **Average Daily Caloric Intake and Total Physical Activity at Baseline and After the 12-Month Intervention **eTable 9. **Mean Heart Rate and Exercise Forms in Aerobic Exercise Group **eFigure. **Office Blood Pressure Distribution
eReferences

Supplement 3.
Data Sharing Statement
