# Supplementary material for: Effect of Tai Chi vs Aerobic Exercise on Blood Pressure in Patients With Prehypertension: A Randomized Clinical Trial
Source: JAMA Netw Open. 2024 Feb 9;7(2):e2354937. doi: 10.1001/jamanetworkopen.2023.54937 (PMC10858403; doi:10.1001/jamanetworkopen.2023.54937)
Supplement: Supplement 1. — Trial Protocol [file jamanetwopen-e2354937-s001.pdf]

Trial Protocol

Effect of Tai Chi vs Aerobic Exercise on Blood Pressure in Patients with Prehypertension: A Randomized Clinical Trial

1. Background

Prehypertension, defined as blood pressure (BP) in the range of 120-139/80-89 mmHg, was introduced by the seventh report of the Joint National Committee on Prevention, Detection, Evaluation, and Treatment of High Blood Pressure in 2003 [1]. Prehypertension is highly prevalent and affects 25-50% of adults worldwide. Compared with optimal BP, the prehypertension increases the risk of incident hypertension, cardiovascular (CV) events and death [2], as it is related to risk factors such as high body mass index, metabolic syndrome, dyslipidemia and impaired glucose metabolism [3,4]. Prehypertension increases the risk of incident hypertension, with annual rates ranging from 8% to 20% in studies lasting 2-4 years, and 4% to 9% in longer-term studies [5,6]. The Guidelines recommend that prehypertension population at low–moderate CV risk should be offered lifestyle advice [7]. Moderate intensity of regular physical activity, as one of the lifestyles, can reduce BP, as well as lower the risk of heart attack and stroke [8].

Aerobic exercise, is recommended as one of the lifestyles that can lower BP [9] and suggested in the guidelines to help both the prevention and treatment of hypertension [7]. However, aerobic exercise has some limitations. The exercise adherence is low and pace and venue also restrict effective training of aerobic exercise. Other useful substitute exercise modes need to be proved effective in reducing BP. As a safe, low-impact, enjoyable, and inexpensive form of exercise, that requires minimal equipment and space, Tai Chi is beneficial to improve exercise adherence and is expected as a viable alternative to aerobic exercise [10,11]. Tai Chi guides people to concentrate on slow and fluid movements, covering all-round adjustments such as balance, core strength, flexibility and so on [12]. Studies have shown that Tai Chi has a positive effect on patients with cardiovascular diseases (CVD) [13], and it can have a beneficial impact on BP when combined with other lifestyle forms changes [14]. Some studies have shown that after 12 weeks, 9 months or 12 months

of intervention, systolic blood pressure (SBP) in the Tai Chi group decreased significantly, suggesting that Tai Chi exercise training can effectively reduce BP in patients with hypertension [15-18].

Therefore, the study aimed to assess the effect of Tai Chi intervention program, compared with aerobic exercise, on the office BP, ambulatory blood pressure monitoring (ABPM) and home blood pressure monitoring (HBPM) in patients with prehypertension. Based on previous studies, Tai Chi was hypothesized to have better effects on lowering BP than aerobic exercise.

2. Study design

This protocol followed the Standard Protocol Items: Recommendations for Interventional Trials (SPIRIT) reporting guideline (Figure 1). The study setting is two hospitals in Beijing, China: Guang'anmen Hospital of China Academy of Chinese Medical Sciences and Dongzhimen Hospital of Beijing University of Chinese Medicine. The study has received approval from the Ethics Committee of two hospitals.

This study is a 12-month, parallel, randomized controlled trial, conducted in two centers. Patients with prehypertension are randomized to one of two intervention groups: 12-month supervised Tai Chi held four times a week, or 12-month supervised aerobic exercise. All groups will be followed up for 12 months.

A one-week induction period is designed for those who meet the inclusion criteria for prehypertension screening; during this period, participants will receive Tai Chi education and train to exclude individuals who cannot tolerate the exercises and improve study compliance. Outcome measurements are collected at baseline, 6 months, and 12 months (Table 1). The staff conducting the BP assessments and the statistician are blinded to treatment tasks and groupings. The study flow chart shows an overview of the study procedure (Figure 2).

Table 1. Sequence of Primary and secondary outcomes measurement during intervention and follow-up

	Baseline	Month 6	Month 12
Time (days)	-7~0	180±14	360±14
**Primary outcome variable**			
SBP in office blood pressure[a]	×	×	×
**Secondary outcome variables**			
DBP in office blood pressure	×	×	×
ABPM[b]	×		×
Caloric assessment of diet[c]	×	×	×
HBPM	×	×	×
IPAQ[d]	×	×	×
SF-36	×		×
Blood taken	×		×
Urine taken	×		×
SCORE system	×		×

[a]Systolic blood pressure (SBP) in office blood pressure is the primary outcome at 12 months; the other collection times are secondary outcome variables. [b]Ambulatory blood pressure monitoring (ABPM) can

provide the average blood pressure estimates during the whole monitoring period, and provide average BP during nighttime and daytime respectively, estimate the variability of BP. [c]Assess the average daily calorie intake of participant for the nearly week. [d]Used to assess the one-week total physical activity including physical activity of occupation, transportation, housework and recreation.

Abbreviations: DBP, diastolic blood pressure; HBPM, home blood pressure monitoring; IPAQ, International Physical Activity Questionnaire; SF-36, Medical Outcome Survey Short-Form 36; SCORE, Systematic COronary Risk Evaluation.

Figure 1. SPIRIT figure. Schedule Of enrolment, interventions, and assessments.

	STUDY PERIOD					
	Enrolment	Allocation	Intervention			Close-out
**TIMEPOINT**	**Week -1**	**0**	**Month 1-5**	**Month 6**	**month 7-11**	**month 12**
**ENROLMENT:**						
**Eligibility screen**	X					
**Informed consent**	X					
**Allocation**		X				
**INTERVENTIONS:**						
**Tai Chi group**			←	—	—	→
**Aerobic exercise group**			←	—	—	→
**ASSESSMENTS:**						
**Baseline characteristics**	X					
**SBP in office blood pressure**	X			X		X
**DBP in office blood pressure**	X			X		X
**ABPM**	X					X
**Caloric assessment of diet**	X			X		X
**HBPM**	X			X		X
**IPAQ**	X			X		X
**SF-36**	X					X
**Blood taken**	X					X
**Urine taken**	X					X
**SCORE system**	X					X
**Adverse events**				X		X

Figure 2. Study flow chart. SBP, systolic blood pressure; DBP, diastolic blood pressure; BP, blood pressure; ABPM, ambulatory blood pressure monitoring; HBPM, home blood pressure monitoring.

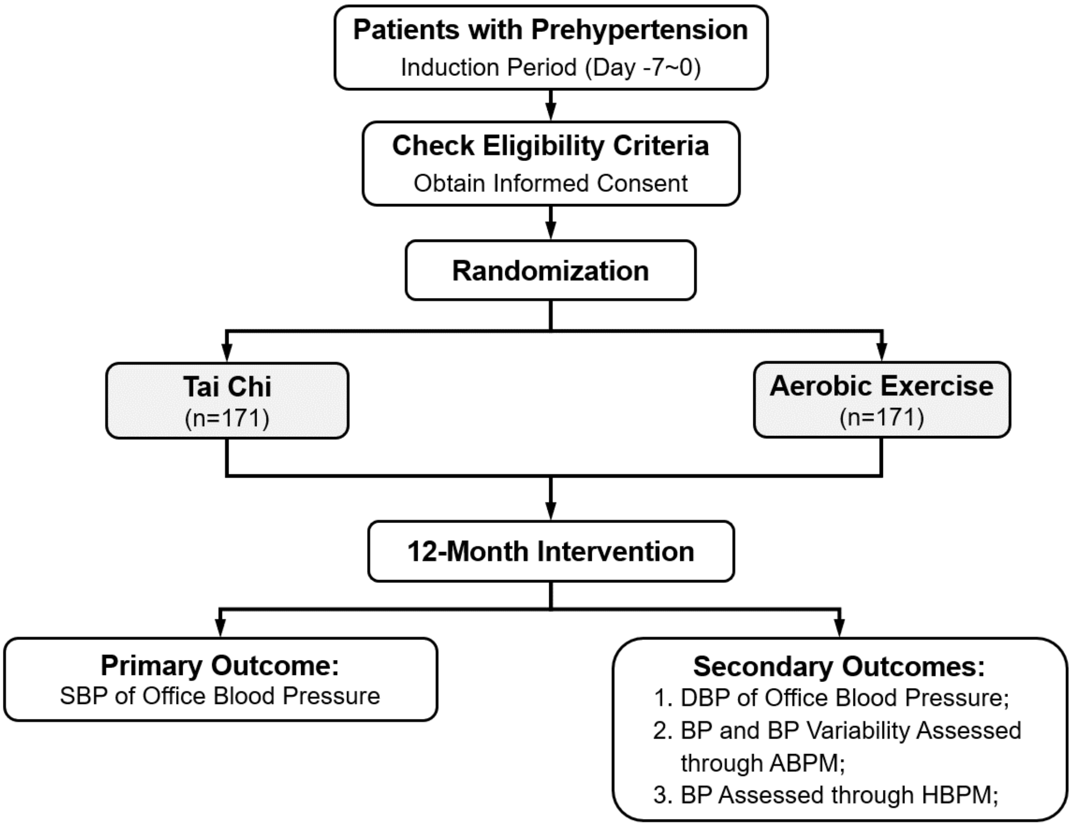

3. Eligibility Criteria

Patients who meet the classification of prehypertension [with a SBP in the range of 120 mmHg to 139 mmHg and/or a diastolic blood pressure (DBP) in the range of 80 mmHg to 89 mmHg] are eligible to participate in this study [1]. Recruitment strategies include the distribution of flyers within the hospital as well as advertisements in print and online media, to ensure adequate enrollment of different study population. Potential participants will be contacted by phone and WeChat to assess whether they meet the basic eligibility criteria for the study. Those who meet the eligibility criteria are invited to come to the hospital for screening, in which case their eligibility criteria are verified. After a complete explanation of the study procedures, each eligible subject who agrees to participate provides informed consent, which is completed by the principal investigator or study coordinator. The study coordinator will inform participants of the schedule of the training sessions, including the date and time.

Participants are considered eligible for this study if they:

(1) are aged from 18 to 65 years;

(2) fulfill the classification of prehypertension: with a SBP in the range of 120 mmHg to 139 mmHg and/or a DBP in the range of 80 mmHg to 89 mmHg [1];

(3) have no western medicine or traditional Chinese medicine, acupuncture and moxibustion were used to control blood pressure (or the treatment was discontinued for 2 weeks);

(4) are willing to be randomized to Tai Chi group or aerobic exercise group;

(5) have ability to complete written questionnaires and operate electronic equipment independently;

(6) are able to give informed consent.

Exclusion criteria are: (1) diagnosed with diabetes mellitus and coronary heart disease;

(2) pregnant and lactating women;

(3) non-dominant arm circumference > 50 cm;

(4) body mass index (BMI) larger than 40.0 kg/m^2;

(5) take benzodiazepines, antipsychotics or oral glucocorticoids (allowed to taken fluoxetine, paroxetine, sertraline, fluvoxamine, citalopram and escitalopram stably within 3 months);

(6) with chronic kidney disease, with eGFR < 60 mL/min;

(7) diagnosed with Shy-Drager syndrome;

(8) alcoholism (male's alcohol intake is more than 25 g/day or 140 g/week or female's alcohol intake is more than 15 g/day or 80 g/week);

(9) has played Tai Chi more than once a month in the past six months;

(10) plays vigorous sports activities more than three times a week;

(11) with musculoskeletal disorder or other disabling diseases lead to the inability to practice Tai Chi or do

aerobic exercise;

(12) current in clinical trials of other drugs or external therapies.

4. Randomization and blinding

Participants, after the induction period, who met all the eligibility criteria and provided written informed consent will be randomly assigned to either Tai Chi or aerobic exercise (control) group, in a 1:1 ratio, with a predicted sample size of 342 patients, 171 in each group. At the same time, the participants are stratified according to SBP of 120–129 mmHg and DBP <80 mmHg, and SBP of 130–139 mmHg or DBP of 80–89 mmHg. At each stratum, the proportion of participants in the Tai Chi and aerobic exercise groups is also randomly assigned in a 1:1 ratio. In order to ensure the concealment of allocation, a 24-hour central web-based automated randomization system is adopted for all randomization processes, using the static random method and the SAS9.4 software PROC PLAN process programming.

When allocation is complete, the outcome assessors who evaluate the effects of the treatments will receive only the participant number, and then interpretation the data under blinded to group allocation. Only outcome assessors are blinded so unblinding will not occur.

5. Study intervention

The maximum waiting time between baseline assessment and interventional therapy is 3 weeks. In order to avoid the influence of seasonal factors on the disease, both Tai Chi and aerobic exercise groups are simultaneously performed. Participants randomly assigned to Tai Chi or aerobic exercise will practice at indoor activity room in or near Guang'anmen Hospital or Dongzhimen Hospital. The number of participants in each class is limited to 20 to ensure the quality of teaching and learning.

- *Lifestyle intervention*

Common interventions are health education and lifestyle guidance. Participants in both groups receive dietary recommendations for weight control and salt intake. DASH eating plan is the best diet that can effectively reduce BP [20]. During the study intervention, participants are advised to follow the DASH diet, which is rich in fruits, vegetables and low-fat dairy foods and with reduced saturated and total fat.

- *Tai Chi intervention*

The 24-form Yang-style Tai Chi consists of 24 standard movements. The four Tai Chi instructors each have extensive experience and will explain and demonstrate Tai Chi principles, practice techniques and safety precautions for each movement at the beginning of the study. The instructor will review these principles and techniques, as throughout the study process and always practice with the participants, to timely and effectively identify and correct the incorrect posture or movement. Participants will also be instructed to concentrate and perform traditional Tai Chi breathing, while performing body movements. Moreover, all four instructors completed the required human subject protection training before the beginning of intervention courses.

Each Tai Chi session will last for 60 min, including 10 min of warm-up exercise, 40 min of Tai Chi teaching and/or practice, and 10 min of relaxation, and occur 4 times a week. Among them, there are no less than twice centralized sessions per week, and for the rest practice, participants can practice at home and upload videos. The instructor could modify and tailor-make for their Tai Chi exercises according to participants' learning and athletic ability. In the initial eighth-week, the participants learn and practice step by step. In each session, participants practice and learn 3 to 4 movements of Tai Chi. After all the 24 Tai Chi forms had been learned (weeks 10 and 11), the Tai Chi instructor (experience > 10 years) will assess the participants. After passing the assessment, the participants will participate in centralized sessions at least once

a week, and practice at home and upload videos for the other three times. Participants are required to sign in to confirm the accurate attendance records, when they attend the Tai Chi session or practice at home. The standard case report forms are used to record and verify the data collected for class attendance, to confirm accurate attendance recordings. The study staff will monitor the participants by monthly home calls throughout the 12-month intervention. Throughout the study, all sessions are regularly monitored and fed back to ensure proper instruction.

- *Aerobic exercise intervention*

Participants randomized to aerobic exercise will receive a supervised, group-format aerobic exercise program. The aerobic exercises training protocol for prehypertension treatment consists of four 60-minute sessions of moderate intensity exercises per week. The aerobic exercises include climbing stairs, jogging, brisk walking and cycling. Each session includes several parts: 10 minutes of warm-up including low-intensity exercise and dynamic stretching; 40 minutes of organized aerobic training, gradually developing from low intensity to medium intensity; 10 minutes cool-down. The training in the sessions is progressive, and all participants gradually increase the duration and intensity of the exercise. In all sessions, instructors will closely monitor to ensure the comfort and safety of participants and to minimize adverse events. Heart rate will be recorded during each session to monitor the intensity of exercise. During the 1-4 weeks, participants are advised to achieve an individualized heart rate of 55% - 65% of estimated maximum heart rate according to their age, and should reach 60% - 70% after 4 weeks. The maximum heart rate is estimated as "$208 - 0.7 \times$ age"[21]. Participants in aerobic exercise group will perform the above exercises 4 times a week, including collective exercises no less than 1 time a week, and the rest 3 times of uploaded videos. A wrist wearable device (HUAWEI band) is used to monitor heart rate. It uses an optical sensor to accurately calculate heart rate through fluctuations in blood flow in the wrist. The data are uploaded to an app, where information is

stored for long time. The data collected for session attendance are recorded using standard case report forms. The study team will contact the participants by monthly phone to monitor their adherence until the 12-month follow-up evaluation. During the study process, all sessions are regularly monitored by the instructors and fed back to ensure correct instruction for the group.

6. Measurements

Referring to the BP measurement method in the Guideline [22], the specific measurement method of the study is as follows. A quiet room is set up for measurement of BP. The upper arm medical electronic sphygmomanometer certified by the internationally accepted protocol is used (Omron HBP-1300). The patients are asked to rest, sitting in a chair, for >10 min, and the first BP measurement is conducted following the rest period. The participant and the researcher should not talk during the rest period or the measurement. The interval of repeated measurements is 1-2 minutes. When the difference between the first two measurements is greater than 10 mmHg, additional measurements are taken. The average of the last two readings is recorded to estimate the individual's level of BP.

Participants receive 24 h-ambulatory blood pressure monitoring (Welch Allyn ABPM 6100). The 24-hour ABPM is programmed to automatically obtain BP records, with the instrument set to obtain readings every 30 minutes throughout the day and every 1 hour at night. ABPM can provide the average BP estimates during the whole monitoring period, and provide average BP during nighttime and daytime respectively, estimate the variability of BP.

Participants are given a free upper-arm cuff device (Lifesense i5S), which enables automatically stores multiple readings, and educated on its use. Referring to the home BP monitoring measurement in the Guideline [7], measurements are taken in a quiet room after 5 min of rest, with the participant seated.

Participants are instructed to obtain home BP measurements two times in the morning after voiding and before eating or vigorous exercise and two times in the evening, with 1 minute apart between readings. Mean home BP is reliable, using the average of two morning and two evening BP readings for at least three consecutive days in one week minimum in each month [23], at the same time, can being used to estimate the variability of BP.

7. Data collection and management

- *Plans for assessment and collection of outcomes*

The researchers shall fill in data to case report forms (CRF) accurately, completely, and timely based on original observations of the subjects. The auditor should monitor whether all CRFs are consistent with the source data and raise questions at any time when any problem occurs. If there are errors and omissions, the researchers should correct them in time.

- *Data management*

All data will initially be entered legibly in the paper CRF. If an error made, it will be crossed through with a single line to ensure that the original entry can still be read. The correct entry will then be inserted clearly. The amendment will be initialed and dated by the person making the correction immediately. It is not permitted to overwrite or use correction fluid. Ensure that paper-based CRF data are securely input into Electronic Data Capture (EDC) system. Access will be restricted to site personnel, trial monitors and data management team. EDC system provides range checks for data values to ensure and improve data quality. Researchers are responsible for ensuring the accuracy of all data entered and recorded in the paper CRF and EDC system.

8. Relevant concomitant care permitted or prohibited during the trial

During the run-in period and the intervention period, antihypertensive drugs or antihypertensive treatment will not be allowed to be used. If the participant has increased BP and cannot tolerate the increased BP, which should be treated immediately, it is not advisable to continue the trial, and the researcher should consider terminating the intervention and switching to another type of clinical treatment.

9. Provisions for post-trial care

Participants will be assessed on a case-by-case basis across all participating sites and will be given the option to remain on the centralized exercise training after completion of the trial if there appears to be a benefit to them. If serious adverse events occur during the study period, the participants are also required to be followed up after the study period. Appropriate measures will be taken to fully protect the interests of participants, such as outpatient or inpatient care or referrals to other specialists.

10. Outcomes and Follow-up

At enrollment, the essential information of participants' sex, age, nation, income, education level, professional characteristics, personal history, family history, height, weight, waist circumference, hip circumference, eating habits and caloric intake are collected. Overall assessment of BP is focused on. Outcomes are assessed at baseline, 6 months, and 12 months (at the end of the intervention).

- *Primary outcome*

The primary outcome is the comparison of SBP of office blood pressure change from baseline to 12 months between Tai Chi group and aerobic exercise group.

- *Secondary outcomes*

BP monitoring methods of office blood pressure, ABPM and HBPM are used at the same time to detect BP

in multiple dimensions. The secondary endpoints are as following:

(1) the comparison of DBP of office blood pressure change from baseline to 12 months between Tai Chi group and aerobic exercise group; (2) the comparison of BP and the variability of BP assessed through ABPM change from baseline to 12 months between Tai Chi group and aerobic exercise group; (3) the comparison of BP assessed through HBPM change from baseline to 12 months between Tai Chi group and aerobic exercise group.

11. Monitoring of Adverse Events

Adverse events must be registered during the study period, which refers to the medical conditions not considered as end points of study. Study participants are monitored for the occurrence of adverse events, during each encounter during the study intervention. A study telephone number is provided to the participants to report adverse events throughout the study. All adverse events are recorded in the case report form during the study intervention.

12. Criteria for study withdrawal

Individuals who decide to no longer participate in the study or who are lost to follow-up (failure to attend the prescheduled visits or cannot be reached by telephone call) can be withdrawn from the study.

13. Confidentiality

Participant individual identification numbers are used to track data collection documents. All data will be kept strictly confidential and only accessed by members of the trial team. Paper CRFs are stored in a locked file cabinet. Access to EDC system is password protected and restricted to the trial team. This protocol, CRFs,

and other documents and materials related to the trial will be kept strictly confidential and will not be disclosed to third parties unless expressly agreed upon by the principal investigator in advance. Staff of the investigators involved in this trial are also bound by the agreement.

14. Statistical Analysis

The data of all participants will be analyzed according to the group to which they were originally assigned, regardless of whether having adhered to the treatment and study procedures. Descriptive statistics, such as the mean (SD) or percentage, are used to summarize baseline characteristics and unadjusted study outcome measures, while assessing intergroup equivalence at baseline. At the end of the study, treatment analysis will be performed according to the protocol. Analysis of variance of continuous variables and χ^2 (or Fisher exact) test for categorical variables are used to compare baseline demographic descriptions and primary and secondary outcome measures of each group. For continuous variables, the differences between the average variations from baseline values and their respective 95% CIs will be calculated. If any characteristic is substantially different at baseline, it will be adjusted in a mixed-model regression analysis. To avoid multiple comparisons, differences between groups will be assessed only if the overall effect of treatment is significant. The source data will be registered in the paper CRF and EDC system, and the pattern of missing data will be evaluated before data analyzing. Missing data will be processed through multiple imputation using a set of baseline characteristics, and 6-month and 12-month results. All analyses will be conducted using IBM SPSS (IBM Corp) or Stata (release 13; StataCorp LP). Level of statistical significance will be set at 0.05.

- *Sample Size*

The sample size calculation was based on the comparison of the office SBP drop of individuals in the Tai Chi group and the aerobic exercise group. According to the average reduction of SBP in the studies conducted

before the start of the trial [15,19], using a conservative estimate, we hypothesized that the SBP in the Tai Chi group would be reduced by 4.6 mmHg more than the aerobic exercise group. Further conservatively assuming an SD of 13.4 of both groups. We estimate a loss of follow-up of 20% and 80% power at a two-sided α level of 0.05. Therefore, 171 participants per group and 342 participants in total is the reasonable sample size of this study. The analyses were performed using PASS version 15.0.

- *Study Outcomes*

The primary outcome measure was the change in office SBP from baseline to 12 months. Secondary outcomes included mean changes in office SBP at 6 months; mean changes in office DBP at 6 and 12 months; mean changes in 24-h Ambulatory BP (24-h ambulatory SBP, 24-h ambulatory DBP, daytime ambulatory SBP, daytime ambulatory DBP, nighttime ambulatory SBP and nighttime ambulatory DBP), BP assessed through HBPM at 12 months; and adverse events (e.g., being hospitalized, or death by any cause), including adverse effects during or after the exercise sessions (e.g., severe hypotension). Other assessments included adherence (assessed via Tai Chi or aerobic exercise sessions) and safety evaluations.

- *Data Set*

Intention-to-treat (ITT) refers to all eligible patients who were randomized to treatment, include drop-out cases. Multiple imputation method will be preferred for analyzing the missing data.

Safety set (SS) refers to the actual data for subjects who receive at least one intervention after randomization and for whom safety indicators are documented. The incidence of adverse reactions is calculated using the number of cases in the safety set.

Adverse events: Will be analyzed, with the number of cases, category, and severity of occurrences counted separately and their relationship to the intervention.

15. Oversight and monitoring

The study group provided coordination and day to day support for the trial. The study leader, Yanwei Xing, supervised the design of the study and will supervise and guide the implementation of the trial. Study leader was responsible for all aspects of local organization, including identifying potential recruits and taking consensus. The trial steering committee is composed of the study leader (Xing), on-site principal investigators of each hospital, and coinvestigators. Questions that arise during the research process will be submitted to the committee for decision-making. Finally, clinical research associates (CRAs) will supervise the study progress at any time and hold a meeting every three months.

The Data Monitoring Committee (DMC) is consisting by a doctor whose major is clinical cardiovascular disease, a scientific researcher whose major is clinical trial methodology and a statistician. DMC is responsible for safety monitoring, reviewing, and evaluating the detailed information of adverse events between groups. According to the evaluation results, DMC could give suggestions to terminate the trial in advance, or take measures to reduce the risk of adverse events and adjust the study protocol. In the middle of the trial, the research team will conduct interim analysis. If the analysis result is consistent with the hypothesis, the test will be continued. If it is inconsistent or even contrary, the expert committee will be consulted to decide whether to continue the test, expand the sample size, or terminate the test.

16. Plans for auditing trial conduct

The monitoring will be conducted by the principal investigator, on-site principal investigators, and DMC every three months, as an audit of trial conduct. Annual progress reports and interim report are provided to the funding agent.

17. Plans for communicating important protocol amendments

Any changes to the protocol will notify the funding agent first then will notify the centers and that a copy of the revised protocol will be added to the Investigator Site File. All subsequent substantial protocol amendments will be documented and submitted to the Ethics Committee for approval before implementation. The principal investigator at each site is responsible for ensuring that all subsequent amendments gain the necessary approval.

18. References

1. Chobanian AV, Bakris GL, Black HR, et al. Seventh report of the Joint National Committee on Prevention, Detection, Evaluation, and Treatment of High Blood Pressure. Hypertension (Dallas, Tex. : 1979) 2003;42(6):1206-52.
2. Egan BM, Stevens-Fabry S. Prehypertension--prevalence, health risks, and management strategies. Nat Rev Cardiol. 2015;12(5):289-300. Epub 2015 Feb 17.
3. Ganguly SS, Al-Shafaee MA, Bhargava K, Duttagupta KK. Prevalence of prehypertension and associated cardiovascular risk profiles among prediabetic Omani adults. BMC public health 2008;8:108.
4. Grotto I, Grossman E, Huerta M, Sharabi Y. Prevalence of prehypertension and associated cardiovascular risk profiles among young Israeli adults. Hypertension (Dallas, Tex. : 1979) 2006;48(2):254-9.
5. Egan BM, Stevens-Fabry S. Prehypertension--prevalence, health risks, and management strategies. Nature reviews. Cardiology 2015;12(5):289-300.
6. Huang Y, Wang S, Cai X, et al. Prehypertension and incidence of cardiovascular disease: a meta-analysis. BMC medicine 2013;11:177.
7. Williams B, Mancia G, Spiering W, et al. 2018 ESC/ESH Guidelines for the management of arterial

hypertension. European heart journal 2018;39(33):3021-104.

8. Grundy SM, Stone NJ, Bailey AL, et al. 2018 AHA/ACC/AACVPR/AAPA/ABC/ACPM/ADA/AGS/APhA/ASPC/NLA/PCNA Guideline on the Management of Blood Cholesterol: A Report of the American College of Cardiology/American Heart Association Task Force on Clinical Practice Guidelines. Circulation 2019;139(25):e1082-e143.

9. Haskell WL, Lee IM, Pate RR, et al. Physical activity and public health: updated recommendation for adults from the American College of Sports Medicine and the American Heart Association. Circulation 2007;116(9):1081-93.

10. Wayne PM, Kaptchuk TJ. Challenges inherent to t'ai chi research: part I--t'ai chi as a complex multicomponent intervention. J Altern Complement Med. 2008 Jan-Feb;14(1):95-102.

11. Wayne PM, Kaptchuk TJ. Challenges inherent to t'ai chi research: part II-defining the intervention and optimal study design. J Altern Complement Med. 2008 Mar;14(2):191-7.

12. Zheng S, Kim C, Lal S, Meier P, Sibbritt D, Zaslawski C. The Effects of Twelve Weeks of Tai Chi Practice on Anxiety in Stressed But Healthy People Compared to Exercise and Wait-List Groups-A Randomized Controlled Trial. Journal of clinical psychology 2018;74(1):83-92.

13. Pan L, Yan J, Guo Y, Yan J. Effects of Tai Chi training on exercise capacity and quality of life in patients with chronic heart failure: a meta-analysis. European journal of heart failure 2013;15(3):316-23.

14. Maris SA, Quintanilla D, Taetzsch A, et al. The combined effects of tai chi, resistance training, and diet on physical function and body composition in obese older women. Journal of aging research 2014;2014:657851.

15. Chan AWK, Chair SY, Lee DTF, et al. Tai Chi exercise is more effective than brisk walking in reducing cardiovascular disease risk factors among adults with hypertension: A randomised controlled trial.

International journal of nursing studies 2018;88:44-52.

16. Ma C, Zhou W, Tang Q, Huang S. The impact of group-based Tai chi on health-status outcomes among community-dwelling older adults with hypertension. Heart & lung: the journal of critical care 2018;47(4):337-44.

17. Sun J, Buys N. Community-Based Mind-Body Meditative Tai Chi Program and Its Effects on Improvement of Blood Pressure, Weight, Renal Function, Serum Lipoprotein, and Quality of Life in Chinese Adults With Hypertension. The American journal of cardiology 2015;116(7):1076-81.

18. Tsai JC, Wang WH, Chan P, et al. The beneficial effects of Tai Chi Chuan on blood pressure and lipid profile and anxiety status in a randomized controlled trial. Journal of alternative and complementary medicine (New York, N.Y.) 2003;9(5):747-54.

19. Wu Y, Johnson BT, Chen S, et al. Tai Ji Quan as antihypertensive lifestyle therapy: A systematic review and meta-analysis. J Sport Health Sci. 2020: S2095-2546(20)30038-7.

20. Schwingshackl L, Chaimani A, Schwedhelm C, et al. Comparative effects of different dietary approaches on blood pressure in hypertensive and pre-hypertensive patients: A systematic review and network meta-analysis. Critical reviews in food science and nutrition 2019;59(16):2674-87.

21. Tanaka H, Monahan KD, Seals DR. Age-predicted maximal heart rate revisited. Journal of the American College of Cardiology 2001;37(1):153-6.

22. Whelton PK, Carey RM, Aronow WS, et al. 2017 ACC/AHA/AAPA/ABC/ACPM/AGS/APhA/ASH/ASPC/NMA/PCNA Guideline for the Prevention, Detection, Evaluation, and Management of High Blood Pressure in Adults: A Report of the American College of Cardiology/American Heart Association Task Force on Clinical Practice Guidelines. Hypertension (Dallas, Tex. : 1979) 2018;71(6):e13-e115.

23. Bello NA, Schwartz JE, Kronish IM, et al. Number of Measurements Needed to Obtain a Reliable Estimate of Home Blood Pressure: Results From the Improving the Detection of Hypertension Study. Journal of the American Heart Association 2018;7(20):e008658.

Statistical Analysis

Effect of Tai Chi vs Aerobic Exercise on Blood Pressure in Patients with Prehypertension: A Randomized Clinical Trial

1. Study Objective

To assess the efficacy of Tai Chi versus aerobic exercise in patients with prehypertension.

2. Study Design

2.1 Sample Size

The sample size calculation was based on the comparison of the office SBP drop of individuals in the Tai Chi group and the aerobic exercise group. According to the average reduction of SBP in the studies conducted before the start of the trial [1-2], using a conservative estimate, we hypothesized that the SBP in the Tai Chi group would be reduced by 4.6 mmHg more than the aerobic exercise group. Further conservatively assuming an SD of 13.4 of both groups. We estimate a loss of follow-up of 20% and 80% power at a two-sided α level of 0.05. Therefore, 171 participants per group and 342 participants in total is the reasonable sample size of this study. The analyses were performed using PASS version 15.0.

2.2 Randomization and Blinding

Participants, after the induction period, who met all the eligibility criteria and provided written informed consent will be randomly assigned to either Tai Chi or aerobic exercise (control) group, in a 1:1 ratio, with a predicted sample size of 342 patients, 171 in each group. At the same time, the participants are stratified according to SBP of 120–129 mmHg and DBP <80 mmHg, and SBP of 130–139 mmHg or DBP of 80–89 mmHg. At each stratum, the proportion of participants in the Tai Chi and aerobic exercise groups is also randomly assigned in a 1:1 ratio. In order to ensure the concealment of allocation, a 24-hour central web-based automated randomisation system is adopted for all randomisation processes, using the static random method and the SAS9.4 software PROC PLAN process programming.

When allocation is complete, the outcome assessors who evaluate the effects of the treatments will receive

only the participant number, and then interpretation the data under blinded to group allocation. Only outcome assessors are blinded so unblinding will not occur.

3. Study Outcomes

The primary outcome measure was the change in office SBP from baseline to 12 months. Secondary outcomes included mean changes in office SBP at 6 months; mean changes in office DBP at 6 and 12 months; mean changes in 24-h Ambulatory BP (24-h ambulatory SBP, 24-h ambulatory DBP, daytime ambulatory SBP, daytime ambulatory DBP, nighttime ambulatory SBP and nighttime ambulatory DBP), BP assessed through HBPM at 12 months; and adverse events (e.g., being hospitalized, or death by any cause), including adverse effects during or after the exercise sessions (e.g., severe hypotension). Other assessments included adherence (assessed via Tai Chi or aerobic exercise sessions) and safety evaluations.

4. Data Set

Intention-to-treat (ITT) refers to all eligible patients who were randomized to treatment, include drop-out cases. Multiple imputation method will be preferred for analyzing the missing data.

Safety set (SS) refers to the actual data for subjects who receive at least one intervention after randomization and for whom safety indicators are documented. The incidence of adverse reactions is calculated using the number of cases in the safety set.

Adverse events: Will be analyzed, with the number of cases, category, and severity of occurrences counted separately and their relationship to the intervention.

5. Statistical Analysis

5.1 General considerations

The data of all participants will be analyzed according to the group to which they were originally assigned, regardless of whether having adhered to the treatment and study procedures. Descriptive statistics, such as the mean (SD) or percentage, are used to summarize baseline characteristics and unadjusted study outcome measures, while assessing intergroup equivalence at baseline. At the end of the study, treatment analysis will be performed according to the protocol. Analysis of variance of continuous variables and χ^2 (or Fisher exact) test for categorical variables are used to compare baseline demographic descriptions and primary and secondary outcome measures of each group. For continuous variables, the differences between the average variations from baseline values and their respective 95% CIs will be calculated. If any characteristic is substantially different at baseline, it will be adjusted in a mixed-model regression analysis. To avoid multiple comparisons, differences between groups will be assessed only if the overall effect of treatment is significant. The source data will be registered in the paper CRF and EDC system, and the pattern of missing data will be evaluated before data analyzing. All analyses will be conducted using IBM SPSS (IBM Corp) or Stata (release 13; StataCorp LP). Level of statistical significance will be set at 0.05.

5.2 Analysis of the primary outcome

The primary outcome is the change (difference) in office SBP from baseline to the end of the 12-month intervention. Mean differences will be expressed with their two-sided 95% confidence interval. Between-group differences in the change from baseline to the end of the 12-month intervention will be tested with Student's independent t-test or the Mann-Whitney U test. Student's paired t-tests or the Wilcoxon signed-rank test will be performed for within-group comparisons from baseline to the end of the 12-month intervention.

5.3 Analysis of the secondary outcomes

Secondary outcomes included mean changes in office SBP at 6 months; mean changes in office DBP at 6 and 12 months; mean changes in 24-h Ambulatory BP (24-h ambulatory SBP, 24-h ambulatory DBP, daytime ambulatory SBP, daytime ambulatory DBP, nighttime ambulatory SBP and nighttime ambulatory DBP), BP assessed through HBPM at 12 months; and adverse events (e.g., being hospitalized, or death by any cause), including adverse effects during or after the exercise sessions (e.g., severe hypotension). The mean change in the secondary outcomes will be analyzed using the same statistical methods as those used for the primary outcome. Between-group differences in the change from baseline to 6 and 12 months will be tested with Student's independent t-test or the Mann-Whitney U test. Student's paired t-tests or the Wilcoxon signed-rank test will be performed for within-group comparisons from baseline to 6 and 12 months.

6. References

1. Chan AWK, Chair SY, Lee DTF, et al. Tai Chi exercise is more effective than brisk walking in reducing cardiovascular disease risk factors among adults with hypertension: A randomised controlled trial. International journal of nursing studies 2018;88:44-52.

2. Wu Y, Johnson BT, Chen S, et al. Tai Ji Quan as antihypertensive lifestyle therapy: A systematic review and meta-analysis. J Sport Health Sci. 2020: S2095-2546(20)30038-7.
