# Supplementary material for: Effect of Tai Chi vs Aerobic Exercise on Blood Pressure in Patients With Prehypertension: A Randomized Clinical Trial
Source: JAMA Netw Open. 2024 Feb 9;7(2):e2354937. doi: 10.1001/jamanetworkopen.2023.54937 (PMC10858403; doi:10.1001/jamanetworkopen.2023.54937)
Supplement: Supplement 2. — eMethods. Criteria for Eligibility, BP Measurement, and Assessments eTable 1. Overview of Exercise Intervention, as per TIDieR Criteria eTable 2. Intervention Fidelity Checklist eTable 3. Baseline Characteristics of Participants Between the 2 Centers eTable 4. Assessment of Differences in the Demographics, BP, and SCORE Between the Patients Who Completed the Study and Those Who Dropped Out eTable 5. Follow-Up Time of the Tai Chi Group and Aerobic Exercise Group eTable 6. Changes in Ambulatory Pulse Rate, Ambulatory Blood Pressure Load, SCORE, and SF-36 After the 12-Month Intervention eTable 7. Changes in Body Composition and Biochemical Parameters After the 12-Month Intervention eTable 8. Average Daily Caloric Intake and Total Physical Activity at Baseline and After the 12-Month Intervention eTable 9. Mean Heart Rate and Exercise Forms in Aerobic Exercise Group eFigure. Office Blood Pressure Distribution eReferences [file jamanetwopen-e2354937-s002.pdf]

Supplementary Online Content

Li X, Chang P, Wu M, et al. Effect of Tai Chi vs Aerobic exercise on blood pressure in patients with prehypertension: a randomized clinical trial. *JAMA Netw Open*. 2024;7(2):e2354937. doi:10.1001/jamanetworkopen.2023.54937

eMethods. Criteria for Eligibility, BP Measurement, and Assessments

eTable 1. Overview of Exercise Intervention, as per TIDieR Criteria

eTable 2. Intervention Fidelity Checklist

eTable 3. Baseline Characteristics of Participants Between the 2 Centers

eTable 4. Assessment of Differences in the Demographics, BP, and SCORE Between the Patients Who Completed the Study and Those Who Dropped Out

eTable 5. Follow-Up Time of the Tai Chi Group and Aerobic Exercise Group

eTable 6. Changes in Ambulatory Pulse Rate, Ambulatory Blood Pressure Load, SCORE, and SF-36 After the 12-Month Intervention

eTable 7. Changes in Body Composition and Biochemical Parameters After the 12-Month Intervention

eTable 8. Average Daily Caloric Intake and Total Physical Activity at Baseline and After the 12-Month Intervention

eTable 9. Mean Heart Rate and Exercise Forms in Aerobic Exercise Group

eFigure. Office Blood Pressure Distribution

eReferences

This supplementary material has been provided by the authors to give readers additional information about their work.

© 2024 Li X et al. *JAMA Network Open*.

eMethods. Criteria for Eligibility, BP Measurement, and Assessments

Detailed criteria for eligibility

Participants are considered eligible for this study if they (1) are aged from 18 to 65 years; (2) fulfill the classification of prehypertension: with an SBP in the range of 120 to 139 mmHg and/or a DBP in the range of 80 mmHg to 89 mmHg;[1] (3) have not been treated with western medicine or traditional Chinese medicine, acupuncture, and moxibustion for blood pressure management (or the treatment was discontinued for two weeks); (4) are willing to be randomized to the Tai Chi group or aerobic exercise group; (5) have the ability to complete written questionnaires and operate electronic equipment independently; and (6) are able to give informed consent.

Exclusion criteria are (1) diagnosed with diabetes mellitus and coronary heart disease; (2) pregnant and lactating women; (3) non-dominant arm circumference > 50 cm; (4) body mass index (BMI) larger than 40.0 kg/m^2; (5) take benzodiazepines, antipsychotics, or oral glucocorticoids (allowed to taken fluoxetine, paroxetine, sertraline, fluvoxamine, citalopram and escitalopram stably within 3 months); (6) with chronic kidney disease, with eGFR < 60 mL/min; (7) diagnosed with Shy-Drager syndrome; (8) alcoholism (male: alcohol intake more than 25 g/day or 140 g/week or female: alcohol intake more than 15 g/day or 80 g/week); (9) has played Tai Chi more than once a month in the past six months; (10) plays vigorous sports activities more than three times a week; (11) with musculoskeletal disorder or other disabling diseases lead to the inability to practice Tai Chi or do aerobic exercise; and (12) currently in clinical trials of other drugs or external therapies.

Measurement of office BP and ambulatory BP

The specific office BP measurement method of the study is as follows. A quiet room was set up for measurement of BP. The upper arm medical electronic sphygmomanometer certified by the internationally accepted protocol was used (Omron HBP-1300). The patients were asked to rest, sitting in a chair, for >10 min, and the first BP measurement is conducted following the rest period. The participant and the researcher should not talk during the rest period or the measurement. The interval of repeated measurements was 1-2 minutes. When the difference between the first two measurements was greater than 10 mmHg, additional measurements were taken. The average of the last two readings was recorded to estimate the individual's level of BP.

The specific ambulatory BP measurement method of the study is as follows. Participants received 24 h-ambulatory blood pressure monitoring (Welch Allyn ABPM 6100). The 24-hour ABPM was programmed to automatically obtain BP records, with the instrument set to obtain readings every 30 minutes throughout the day and every 1 hour at night. Ambulatory BP

© 2024 Li X et al. *JAMA Network Open*.

monitoring (ABPM) could provide the average BP estimates during the whole monitoring period and provide average BP during nighttime and daytime respectively. The effective number of BP readings was at least 20 throughout the day and 8 at night, viewed as a completed ABPM.

Detailed methods of SF-36, SCORE, Calorie assessment of diet and IPAQ

(1) The physical and mental health and physical function scale scores were calculated from the medical outcomes study 36-item Short-Form Health Survey (SF-36)[3], a standard health-related quality-of-life assessment. The SF-36 comprises eight domains: General Health, Mental Health, Bodily Pain, Physical Functioning, Social Functioning, Role Emotional, Role Physical, and Vitality. Each of the eight SF-36 domains was calculated with a range of 0 (worst) to 100 (best).

(2) The Systemic Coronary Risk Estimation (SCORE) system estimates an individual's 10-year risk of fatal cardiovascular disease.[4, 5] Our trial used the low-risk SCORE chart to estimate the total risk in apparently healthy individuals.

(3) At baseline and 12 months, participants reported their food intake over the past 7 days. We assessed the average daily calorie intake of participant for the nearly week.

(4) One-week total physical activity was assessed using the long-form International Physical Activity Questionnaire (IPAQ)[6] and calculated as the total of occupation, transportation, housework, and recreational activity reported in metabolic equivalents (MET) × min/week.

Physical activity was categorized into recreational and non-recreational physical activities (occupational, transportation, and housework). Total physical activity was categorized as low (<600 MET × min/week), moderate (600–3000 MET × min/week), and high (>3000 MET × min/week), corresponding to less than 150 min/week, 150–750 min/week, and more than 750 min/week of moderate-intensity physical activity.

© 2024 Li X et al. *JAMA Network Open*.

eTable 1. Overview of Exercise Intervention, as per TIDieR Criteria

Item number	TIDieR Item	Description
	**BRIEF NAME**	
1	Provide the name or a phrase that describes the intervention.	Tai Chi and aerobic exercise.
	**WHY**	
2	Describe any rationale, theory, or goal of the elements essential to the intervention.	Exercise interventions reduce blood pressure in individuals with hypertension or prehypertension. early and effective intervention and treatment should be highly supported for the prehypertension population to adjust the prevention strategy and reduce the occurrence of future hypertension and organ damage.
	**WHAT**	
3	Materials: Describe any physical or informational materials used in the intervention, including those provided to participants or used in intervention delivery or in training of intervention providers. Provide information on where the materials can be accessed (e.g. online appendix, URL).	Every trial participant in the exercise arm is given an exercise diary.
4	Procedures: Describe each of the procedures, activities, and/or processes used in the intervention, including any enabling or support activities.	Tai Chi intervention<br>The 24-form Yang-style Tai Chi consists of 24 standard movements. Participants were instructed to concentrate and perform traditional Tai Chi breathing, while performing body movements. Each Tai Chi session lasted for 60 min, including 10 min of warm-up exercise, 40 min of Tai Chi teaching and/or practice, and 10 min of relaxation, and occurred 4 times a week. The instructor modified and tailor-made for their Tai Chi exercises according to participants' learning and athletic ability. In the initial eighth-week, the participants learned and practiced step by step. In each session, participants practiced and learned 3 to 4 movements of Tai Chi.<br>Aerobic exercise intervention<br>Participants randomized to aerobic exercise received a supervised, group-format aerobic exercise program. The aerobic exercises training protocol for prehypertension treatment consisted of four 60-minute sessions of moderate intensity exercises per week. The aerobic exercises included climbing stairs, jogging, brisk walking and cycling. Each session included several parts: 10 minutes of warm-up including low-intensity exercise and dynamic stretching; 40 minutes of organized aerobic training, gradually developing from low intensity to medium intensity; 10 minutes cool-down. The training in the sessions

© 2024 Li X et al. *JAMA Network Open*.

		was progressive, and all participants gradually increased the duration and intensity of the exercise.
	**WHO PROVIDED**	
5	For each category of intervention provider (e.g. psychologist, nursing assistant), describe their expertise, background and any specific training given.	Tai Chi intervention<br>The four Tai Chi instructors each had extensive experience and explain ed and demonstrated Tai Chi principles, practice techniques and safety precautions for each movement at the beginning of the study. The instructor reviewed these principles and techniques, as throughout the study process and always practice with the participants, to timely and effectively identify and correct the incorrect posture or movement. After all the 24 Tai Chi forms had been learned (weeks 10 and 11), the Tai Chi instructor (experience > 10 years) assessed the participants.<br>Aerobic exercise intervention<br>Aerobic exercise group was equipped with two professional sports instructors. In all sessions, instructors closely monitored to ensure the comfort and safety of participants and to minimize adverse events. During the study process, all sessions were regularly monitored by the instructors and fed back to ensure correct instruction for the group.
	**HOW**	
6	Describe the modes of delivery (e.g. face-to-face or by some other mechanism, such as internet or telephone) of the intervention and whether it was provided individually or in a group.	Tai Chi intervention<br>The mode of delivery was face-to-face in the initial nine-week provided in a group. After all the 24 Tai Chi forms had been learned (weeks 10 and 11), the Tai Chi instructor (experience > 10 years) assessed the participants. After passing the assessment, the participants participated in centralized sessions at least once a week, and practiced at home and uploaded videos for the other three times provided individually.<br>Aerobic exercise intervention<br>Participants in aerobic exercise group performed 4 times a week, including collective exercises no less than 1 time a week, and the rest 3 times of uploaded videos provided individually.
	**WHERE**	
7	Describe the type(s) of location(s) where the intervention occurred, including any necessary infrastructure or relevant features.	In the case of collective exercises, participants randomly assigned to Tai Chi or aerobic exercise practiced at indoor activity room in or near Guang'anmen Hospital or Dongzhimen Hospital. In the case of uploading videos, the exercise program was conducted independently at the participant's home.
	**WHEN and HOW MUCH**	
8	Describe the number of times the intervention was delivered and over what period of time including the number of sessions, their schedule, and their duration, intensity or dose.	Tai Chi intervention<br>Each Tai Chi session lasted for 60 min, including 10 min of warm-up exercise, 40 min of Tai Chi teaching and/or practice, and 10 min of relaxation, and occur 4 times a week. Among them, there were no less than twice centralized sessions per week, and for the rest practice, participants can practice at home and upload videos. The instructor could modify and tailor-make for their Tai Chi exercises according to

© 2024 Li X et al. *JAMA Network Open*.

		participants' learning and athletic ability. In the initial eighth-week, the participants learned and practiced step by step. In each session, participants practiced and learned 3 to 4 movements of Tai Chi. After all the 24 Tai Chi forms had been learned (weeks 10 and 11), the Tai Chi instructor (experience > 10 years) assessed the participants.<br>Aerobic exercise intervention<br>Each session included several parts: 10 minutes of warm-up including low-intensity exercise and dynamic stretching; 40 minutes of organized aerobic training, gradually developing from low intensity to medium intensity; 10 minutes cool-down. The training in the sessions was progressive, and all participants gradually increase the duration and intensity of the exercise. Heart rate was recorded during each session to monitor the intensity of exercise. During the 1-4 weeks, participants were advised to achieve an individualized heart rate of 55% - 65% of estimated maximum heart rate according to their age, and should reach 60% - 70% after 4 weeks. The maximum heart rate was estimated as "208 − 0.7 × age".
	**TAILORING**	
9	If the intervention was planned to be personalised, titrated or adapted, then describe what, why, when, and how.	Exercise progression (frequency, level, and sets) was a joint-decision making process, depending on current progress and ability. Type and level of aerobic exercise varied by participant.
	**MODIFICATIONS**	
10	If the intervention was modified during the course of the study, describe the changes (what, why, when, and how).	Not applicable.
	**HOW WELL**	
11	Planned: If intervention adherence or fidelity was assessed, describe how and by whom, and if any strategies were used to maintain or improve fidelity, describe them.	Participants were asked to complete and return exercise diaries to record type and number of exercises performed over the duration of the study. The study team contacted the participants by monthly phone to monitor their adherence until the 12-month follow-up evaluation. The instructors reviewed the exercise diary with the patient at session to monitor adherence and review progress. Throughout the study, all sessions were regularly monitored and fed back.
12	Actual: If intervention adherence or fidelity was assessed, describe the extent to which the intervention was delivered as planned.	The overall mean attendance rates of the Tai Chi and aerobic exercise groups during the 12 months of intervention were 87.3% and 85.7%, respectively.

© 2024 Li X et al. *JAMA Network Open*.

eTable 2. Intervention Fidelity Checklist

**Fidelity of Tai Chi intervention**	
**Item**	**Strategies**
Requirement of interveners	
	• All Tai Chi sessions were conducted by experienced and qualified Tai Chi instructors.<br>• The instructors practiced with the participants, to timely and effectively identify and correct the incorrect posture or movement.<br>• In all sessions, instructors closely monitored to ensure the comfort and safety of participants.<br>• During the study process, all sessions were regularly monitored by the instructors and fed back to ensure correct instruction for the group.
Implementation of intervention	
	• The Tai Chi sessions taught the 24-form Yang-style Tai Chi.<br>• Participants were instructed to concentrate and perform traditional Tai Chi breathing, while performing body movements.<br>• After all the 24 Tai Chi forms had been learned (weeks 10 and 11), the Tai Chi instructor (experience > 10 years) assessed the participants.
Participant compliance	
	• The standard case report forms were used to record and verify the data collected for class attendance, to confirm accurate attendance recordings.<br>• The study team contacted the participants by monthly phone to monitor their adherence until the 12-month follow-up evaluation.
**Fidelity of aerobic exercise intervention**	
**Item**	**Strategies**
Requirement of interveners	
	• During the 1-4 weeks, participants were advised to achieve an individualized heart rate of 55% - 65% of estimated maximum heart rate according to their age, and should reach 60% - 70% after 4 weeks. The maximum heart rate was estimated as "208 − 0.7 × age".<br>• The instructors practiced with the participants, to timely and effectively identify and correct the incorrect posture or movement.<br>• In all sessions, instructors closely monitored to ensure the comfort and safety of participants.<br>• During the study process, all sessions were regularly monitored by the instructors and fed back to ensure correct instruction for the group.
Implementation of intervention	
	• The aerobic exercises included climbing stairs, jogging, brisk walking and cycling.<br>• Heart rate was recorded during each session to monitor the intensity of exercise.
Participant compliance	
	• The standard case report forms were used to record and verify the data collected for class attendance, to confirm accurate attendance recordings.

© 2024 Li X et al. *JAMA Network Open*.

- The study team contacted the participants by monthly phone to monitor their adherence until the 12-month follow-up evaluation.

eTable 3. Baseline Characteristics of Participants Between the 2 Centers

**Characteristic**	**GAM (n = 238)**	**DZM (n = 104)**	***P* Value**
Age, y	51.1 (11.2)	45.0 (12.3)	0.000
Female, n (%)	126 (52.9)	50 (48.1)	0.408
Male, n (%)	112 (47.1)	54 (51.9)	0.408
Weight, kg	69.9 (11.5)	70.0 (12.3)	0.952
Body mass index*, kg/m²	25.3 (3.3)	24.9 (3.2)	0.406
Waist circumference, cm	88.0 (9.3)	88.0 (9.7)	0.980
**Medical history, n (%)**			
Current smoking	23 (9.7)	16 (15.4)	0.126
Previous smoker	11 (4.6)	2 (1.9)	0.372
Hyperlipidemia	38 (16.0)	30 (28.8)	0.006
Family history of hypertension	156 (65.5)	82 (78.8)	0.014
Family history of coronary artery disease	74 (31.1)	37 (35.6)	0.415
SCORE†	1.16 (1.58)	0.43 (0.87)	0.000
**Office BP, mm Hg**			
SBP	132.8 (6.3)	131.8 (5.3)	0.192
DBP	84.1 (5.1)	84.8 (4.0)	0.193
**Ambulatory BP, mm Hg**			
24-h SBP	129.1 (9.4)	127.2 (9.5)	0.091
24-h DBP	81.4 (8.3)	82.1 (8.3)	0.443
Daytime SBP	131.6 (9.4)	130.2 (9.6)	0.194
Daytime DBP	82.7 (8.6)	83.6 (8.7)	0.413
Nighttime SBP	123.9 (14.1)	121.6 (11.5)	0.146
Nighttime DBP	78.8 (10.8)	78.9 (10.3)	0.885
Fasting plasma glucose, mmol/L	5.81 (1.27)	5.29 (0.61)	0.000
HbA1c, %	5.83 (0.47)	5.39 (0.35)	0.000
Total cholesterol, mmol/L	5.28 (1.02)	4.81 (0.81)	0.000
Triglycerides, mmol/L	1.79 (2.16)	1.68 (1.01)	0.600
LDL cholesterol, mmol/L	3.23 (0.73)	2.93 (0.66)	0.000
HDL cholesterol, mmol/L	1.38 (0.32)	1.23 (0.25)	0.000
Creatinine, µmol/L	63.83 (12.39)	62.57 (12.79)	0.396

Data are shown as mean (SD) or n (%). Abbreviations: GAM, Guang'anmen Hospital of China Academy of Chinese

Medical Sciences; DZM, Dongzhimen Hospital of Beijing University of Chinese Medicine; SBP, systolic blood pressure; DBP, diastolic blood pressure; LDL, low-density lipoprotein; HbA1c, glycated hemoglobin; HDL, high-density lipoprotein; SCORE, Systematic Coronary Risk Evaluation.

*Calculated as weight in kilograms divided by height in meters squared.

†The SCORE system estimates an individual's 10-y risk of fatal cardiovascular disease.

© 2024 Li X et al. *JAMA Network Open*.

eTable 4. Assessment of Differences in the Demographics, BP, and SCORE Between the Patients Who Completed the Study and Those Who Dropped Out

	**Patients completed the study (n = 283)**	**Patients dropped out (n = 59)**	***P* Value**
Age, y	50.1 (11.7)	45.3 (12.1)	0.005
Female, No. (%)	153 (54.1)	23 (39.0)	0.035
Male, No. (%)	130 (45.9)	36 (61.0)	0.035
Weight, kg	69.6 (11.7)	71.9 (11.7)	0.168
Body mass index, kg/m²	25.1 (3.3)	25.3 (3.0)	0.742
Waist circumference, cm	87.6 (9.3)	89.7 (10.0)	0.123
**Medical history, No. (%)**			
Previous smoker	28 (9.9)	9 (15.3)	0.228
Hyperlipidemia	58 (20.5)	10 (16.9)	0.535
Family history of hypertension	199 (70.3)	39 (66.1)	0.522
Family history of coronary artery disease	90 (31.8)	21 (35.6)	0.572
SCORE	0.97 (1.42)	0.81 (1.56)	0.448
**Office BP, mm Hg**			
SBP	132.6 (6.0)	132.1 (6.3)	0.542
DBP	84.1 (5.0)	85.1 (3.4)	0.184
**Ambulatory BP, mm Hg**			
24-h SBP	128.4 (9.4)	129.0 (10.2)	0.698
24-h DBP	81.3 (8.0)	83.2 (9.4)	0.109
Daytime SBP	131.1 (9.3)	131.6 (10.2)	0.720
Daytime DBP	82.5 (8.3)	85.2 (9.8)	0.035
Nighttime SBP	123.1 (13.4)	123.8 (13.4)	0.712
Nighttime DBP	78.7 (10.2)	79.5 (12.7)	0.571

Data are shown as mean (SD) or n (%). Abbreviations: SBP, systolic blood pressure; DBP, diastolic blood pressure; SCORE, Systematic Coronary Risk Evaluation.

© 2024 Li X et al. *JAMA Network Open*.

eTable 5. Follow-Up Time of the Tai Chi Group and Aerobic Exercise Group

	Tai Chi Group	Aerobic Exercise Group	*P* Value
Follow-up, median (IQR), months	12.0 (11.93-12.13)	12.0 (11.77-12.15)	0.129

Data are shown as median (IQR).

© 2024 Li X et al. *JAMA Network Open*.

eTable 6. Changes in Ambulatory Pulse Rate, Ambulatory Blood Pressure Load, SCORE, and SF-36 After the 12-Month Intervention

	Tai Chi Group (n = 173)			Aerobic Exercise Group (n = 169)				
	**Baseline**	**12 months**	**Change from baseline**	**Baseline**	**12 months**	**Change from baseline**	**Between-Group Difference in Change**	***P* Value**
**Ambulatory pulse rate, beats/min**								
24-h	74.2 (8.1)	72.1 (7.4)	-2.09 (6.56) ‡	73.6 (7.6)	72.4 (6.9)	-1.24 (6.82) *	-0.85 (-2.28 to 0.57)	0.240
Daytime	77.8 (9.0)	75.8 (8.0)	-1.99 (7.39) †	78.0 (8.4)	76.4 (7.6)	-1.61 (8.21) *	-0.38 (-2.05 to 1.28)	0.651
Nighttime	67.3 (9.0)	64.8 (7.4)	-2.56 (7.88) ‡	65.4 (9.3)	65.1 (6.5)	-0.31 (8.10)	-2.25 (-3.95 to -0.55)	0.010
**Ambulatory BP load§, %**								
24-h SBP load	42.6 (23.5)	34.5 (18.0)	-8.11 (23.01) ‡	42.0 (24.1)	40.0 (19.7)	-1.98 (20.81)	-6.13 (-10.80 to -1.45)	0.010
24-h DBP load	38.2 (23.9)	33.2 (20.1)	-4.91 (22.88) †	37.0 (26.0)	32.7 (20.7)	-4.30 (21.78) *	-0.61 (-5.37 to 4.15)	0.801
Daytime SBP load	34.7 (24.7)	26.5 (18.2)	-8.26 (23.18) ‡	34.2 (25.5)	29.4 (19.8)	-4.76 (24.36) *	-3.50 (-8.57 to 1.56)	0.175
Daytime DBP load	32.2 (25.1)	27.8 (20.5)	-4.37 (23.60) *	31.8 (26.7)	26.5 (21.0)	-5.36 (23.80) †	0.98 (-4.06 to 6.03)	0.701
Nighttime SBP load	58.6 (28.7)	50.7 (24.8)	-7.87 (31.91) †	57.5 (30.0)	59.3 (26.3)	1.80 (23.45)	-9.67 (-15.65 to -3.70)	0.002
Nighttime DBP load	48.6 (29.7)	44.8 (24.6)	-3.79 (31.60)	47.0 (30.4)	43.8 (24.5)	-3.20 (25.94)	-0.59 (-6.76 to 5.57)	0.850
SCORE	0.8 (1.1)	0.8 (1.0)	-0.06 (0.51)	1.1 (1.7)	1.0 (1.4)	-0.03 (0.60)	-0.03(-0.15 to 0.09)	0.602
**SF-36**								
General Health	68.0 (21.7)	73.0 (15.5)	5.02 (23.03) †	68.1 (19.0)	71.0 (15.8)	2.84 (20.14)	2.18 (-2.43 to 6.79)	0.352
Mental Health	81.7 (15.8)	85.9 (12.1)	4.18 (15.01) ‡	81.0 (16.4)	82.7 (16.2)	1.71 (17.79)	2.47 (-1.04 to 5.97)	0.167
Bodily Pain	83.7 (17.8)	84.8 (17.4)	1.07 (19.58)	82.8 (19.3)	82.0 (20.5)	-0.80 (22.21)	1.87 (-2.59 to 6.33)	0.410

© 2024 Li X et al. *JAMA Network Open*.

Physical Functioning	96.2 (5.2)	96.1 (6.1)	-0.11 (6.99)	96.0 (5.3)	95.4 (7.2)	-0.58 (6.93)	0.47 (-1.02 to 1.95)	0.536
Social Functioning	93.6 (12.2)	96.4 (8.2)	2.75 (11.29) †	91.0 (13.5)	92.4 (13.6)	1.37 (14.65)	1.38 (-1.41 to 4.16)	0.332
Role Emotional	85.5 (31.9)	90.7 (23.2)	5.24 (32.31) *	86.6 (30.9)	85.6 (29.8)	-1.01 (39.59)	6.25 (-1.44 to 13.94)	0.111
Role Physical	92.4 (23.3)	94.3 (19.7)	1.86 (24.53)	90.4 (27.8)	90.4 (24.1)	-0.03 (29.76)	1.89 (-3.92 to 7.70)	0.522
Vitality	79.7 (16.5)	82.6 (12.5)	2.89 (14.36) †	78.8 (15.6)	80.9 (14.0)	2.09 (16.58)	0.80 (-2.50 to 4.10)	0.634

Data are shown as mean (SD) or mean (95% CI). Abbreviations: BP, blood pressure; SBP, systolic blood pressure; DBP, diastolic blood pressure; SF-36, medical outcomes study 36-item Short-Form Health Survey.

*Significantly different from baseline to post-intervention, *P* <0.05.

†Significantly different from baseline to post-intervention, *P* <0.01.

‡ Significantly different from baseline to post-intervention, *P* <0.001.

§ BP load was defined as the percentage of readings above the 95th percentile. SBP and DBP load values were calculated from the 24-hour ambulatory blood pressure monitoring analysis.

© 2024 Li X et al. *JAMA Network Open*.

eTable 7. Changes in Body Composition and Biochemical Parameters After the 12-Month Intervention

	Tai Chi Group (n = 173)	Aerobic Exercise Group (n = 169)	Between-group mean difference (95% CI)	*P* Value
Weight, kg	-0.38 (4.66)	-1.07 (5.80)	0.69 (-0.43 to 1.81)	0.224
Body mass index, kg/m²	-0.18 (1.61)	-0.31 (1.82)	0.13 (-0.23 to 0.50)	0.477
Waist circumference, cm	-1.49 (5.16) ‡	-1.64 (6.24) †	0.14 (-1.07 to 1.36)	0.816
**Lipid, and metabolic parameters**				
Fasting plasma glucose, mmol/L	0.05 (0.80)	0.10 (1.11)	-0.05 (-0.25 to 0.16)	0.651
HbA1c, %	0.02 (0.42)	-0.02 (0.22)	0.04 (-0.03 to 0.11)	0.271
Total cholesterol, mmol/L	0.01 (0.69)	0.05 (0.80)	-0.03 (-0.19 to 0.12)	0.666
Triglycerides, mmol/L	-0.10 (1.03)	-0.22 (2.13)	0.13 (-0.23 to 0.48)	0.477
LDL cholesterol, mmol/L	-0.03 (0.53)	0.06 (0.67)	-0.10 (-0.23 to 0.03)	0.136
HDL cholesterol, mmol/L	0.04 (0.21)	0.04 (0.22)	0.01 (-0.04 to 0.05)	0.811
Creatinine, µmol/L	2.53 (7.02)	1.76 (5.98)	0.78 (-0.61 to 2.17)	0.273

Data are shown as mean (SD) or mean (95% CI). Abbreviations: LDL, low-density lipoprotein; HbA1c, glycated hemoglobin; HDL, high-density lipoprotein.

†Significantly different from baseline to post-intervention, *P* <0.01.

‡ Significantly different from baseline to post-intervention, *P* <0.001.

© 2024 Li X et al. *JAMA Network Open*.

eTable 8. Average Daily Caloric Intake and Total Physical Activity at Baseline and After the 12-Month Intervention

	Baseline			12 months		
	**Tai Chi Group**	**Aerobic Exercise Group**	***P* Value**	**Tai Chi Group**	**Aerobic Exercise Group**	***P* Value**
**Calorie assessment of diet**						
Average daily caloric intake, median (IQR), kcal/day	2365.0 (2200.0-2600.0)	2400.0 (2200.0-2607.5)	0.590	2400.0 (2100.0-2600.0)	2400.0 (2100.0-2650.0)	0.405
**IPAQ**						
Total physical activity, median (IQR), MET × min/week	2661.0 (1386.0-4403.3)	3073.5 (1882.5-4527.0)	0.176	3039.0 (1777.5-4786.8)	3264.0 (1823.0-5049.5)	0.570
Low physical activity*, n (%)	14 (9.9%)	11(7.8%)	/	7 (4.9%)	9 (6.4%)	/
Moderate physical activity†, n (%)	67 (47.2%)	59 (41.8%)	/	61(43.0%)	56 (39.7%)	/
High physical activity‡, n (%)	61 (43.0%)	71 (50.4%)	/	74 (52.1%)	76 (53.9%)	/

Data are shown as median (IQR) or n (%). Abbreviations: IQR, interquartile range; IPAQ, International Physical Activity Questionnaire; MET, metabolic equivalents.

* Low physical activity <600 MET × min/week or <150 min/week of moderate-intensity physical activity.

† Moderate physical activity 600–3000 MET × min/week or 150–750 min/week of moderate-intensity physical activity.

‡ High physical activity >3000 MET × min/week or >750 min/week of moderate-intensity physical activity.

© 2024 Li X et al. *JAMA Network Open*.

eTable 9. Mean Heart Rate and Exercise Forms in Aerobic Exercise Group

	**Aerobic Exercise Group**
Mean heart rate, beats/min	114.2 (8.25)
**Aerobic exercise forms**	
Brisk walking, n (%)	48 (34.0)
Jogging, n (%)	19 (13.5)
Combining brisk walking with jogging, n (%)	28 (19.9)
Combining brisk walking with climbing stairs, n (%)	23 (16.3)
Combining brisk walking with cycling, n (%)	5 (3.5)
Combining jogging with cycling, n (%)	5 (3.5)
Combining jogging with climbing stairs, n (%)	7 (5.0)
Combining climbing stairs with cycling, n (%)	6 (4.3)

Data are shown as mean (SD) or n (%).

© 2024 Li X et al. *JAMA Network Open*.

eFigure. Office Blood Pressure Distribution

The distribution of the office blood pressure of different stratification at baseline and after 12-month intervention in Tai Chi and aerobic exercise groups. Abbreviations: BP, blood pressure.

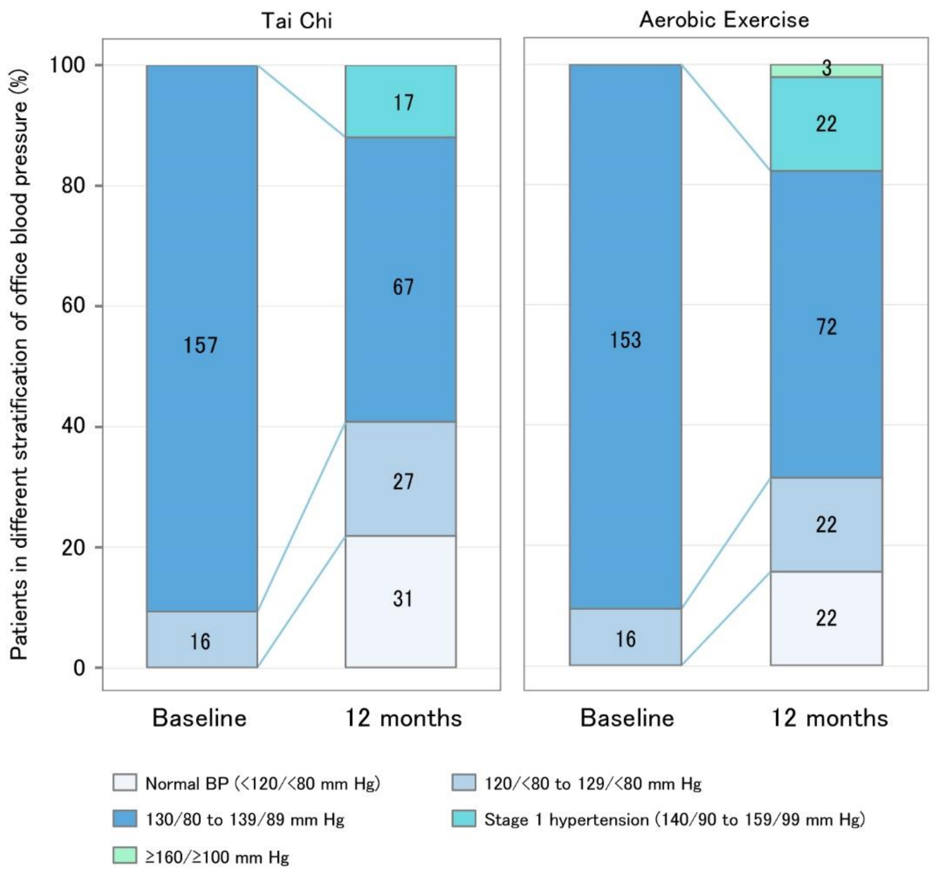

eFigure

© 2024 Li X et al. *JAMA Network Open*.

eReferences

1. Chobanian AV, Bakris GL, Black HR, et al. Seventh report of the Joint National Committee on Prevention, Detection, Evaluation, and Treatment of High Blood Pressure. *Hypertension*. 2003;42(6):1206-52. doi: 10.1161/01.HYP.0000107251.49515.c2

2. Mena L, Pintos S, Queipo NV, et al. A reliable index for the prognostic significance of blood pressure variability. *J Hypertens*. 2005;23(3):505-11. doi: 10.1097/01.hjh.0000160205.81652.5a

3. Ware JE, Jr., Sherbourne CD. The MOS 36-item short-form health survey (SF-36). I. Conceptual framework and item selection. *Med Care*. 1992;30(6):473-83.

4. Conroy RM, Pyörälä K, Fitzgerald AP, et al. Estimation of ten-year risk of fatal cardiovascular disease in Europe: the SCORE project. *Eur Heart J*. 2003;24(11):987-1003. doi: 10.1016/s0195-668x(03)00114-3

5. Piepoli MF, Hoes AW, Agewall S, et al. 2016 European Guidelines on cardiovascular disease prevention in clinical practice: The Sixth Joint Task Force of the European Society of Cardiology and Other Societies on Cardiovascular Disease Prevention in Clinical Practice (constituted by representatives of 10 societies and by invited experts): Developed with the special contribution of the European Association for Cardiovascular Prevention & Rehabilitation (EACPR). *Eur J Prev Cardiol*. 2016;23(11):Np1-np96. doi: 10.1177/2047487316653709

6. Craig CL, Marshall AL, Sjöström M, et al. International physical activity questionnaire: 12-country reliability and validity. *Med Sci Sports Exerc*. 2003;35(8):1381-95. doi: 10.1249/01.mss.0000078924.61453.fb

© 2024 Li X et al. *JAMA Network Open*.
