# Supplementary material for: Effect of Tai Chi vs Aerobic Exercise on Blood Pressure in Patients With Prehypertension: A Randomized Clinical Trial
Source: JAMA Netw Open. 2024 Feb 9;7(2):e2354937. doi: 10.1001/jamanetworkopen.2023.54937 (PMC10858403; doi:10.1001/jamanetworkopen.2023.54937)
Supplement: Supplement 3. — Data Sharing Statement [file jamanetwopen-e2354937-s003.pdf]

Data Sharing Statement

Li. Effect of Tai Chi vs Aerobic Exercise on Blood Pressure in Patients With Prehypertension. *JAMA Netw Open*. Published February 09, 2024. doi:10.1001/jamanetworkopen.2023.54937

Data
Data available: Yes
Data types: Deidentified participant data
How to access data: xingyanwei12345@163.com
When available: beginning date: 12-31-2024

Supporting Documents
Document types: None

Additional Information
Who can access the data: The data will be available to researchers who have been approved for a specified purpose and will sign a data access agreement with the relevant parties.
Types of analyses: Individual participant data that underlie the results of this article, after deidentification, will be available for researchers who provide a methodologically sound proposal (e.g. individual participant meta-analysis).
Mechanisms of data availability: Data will be made available after the approval of a proposal.
